# Self-Contained Lateral-Flow Microfluidic Bead-Based Assay for Rapid Quantification of Early-Stage Kidney Biomarkers

**DOI:** 10.1021/acs.analchem.6c01144

**Published:** 2026-07-01

**Authors:** Gloria Porro, Micaela Siria Cristofori, Céline Gagnieux, Elia Pennati, Pierre-Emmanuel Thiriet, Daniel Constam, Carlotta Guiducci

**Affiliations:** † Laboratory of Life Science Electronics, 27218 École Polytechnique Fédérale de Lausanne, Lausanne 1015, Switzerland; ‡ Laboratory of Developmental and Cancer Cell Biology, École Polytechnique Fédérale de Lausanne, Lausanne 1015, Switzerland; § Rea Diagnostics SA, Lausanne 1015, Switzerland

## Abstract

Kidney diseases, whether acute or chronic, remain among the most challenging conditions to diagnose due to their complex and often asymptomatic progression. Emerging biomarkers such as neutrophil gelatinase-associated lipocalin (NGAL) and cystatin C (CysC) correlate with early pathogenesis and offer complementary insights into disease etiology. However, quantification of their levels, which is essential for patient staging, is available only in centralized laboratories. We present the lateral-flow microfluidic bead-based assay (LMBA), the first rapid, self-contained system for NGAL and CysC analysis. LMBA integrates a microfluidic chip with paper-based components to run automated high-resolution immunoassays in 20 min. Its modular architecture, decoupling reagent storage and flow control from assay binding and readout, offers significant design flexibility. Validated against ELISA, LMBA exhibits comparable sensitivity with an extended dynamic range, requiring only 1 μL of the original blood or urine sample prior to dilution. We applied the LMBA to neonatal mouse models of polycystic kidney disease and measured biomarker elevations consistent with cyst progression. This scalable platform supports preclinical research and holds strong potential for decentralized kidney disease diagnostics.

## Graphical abstract

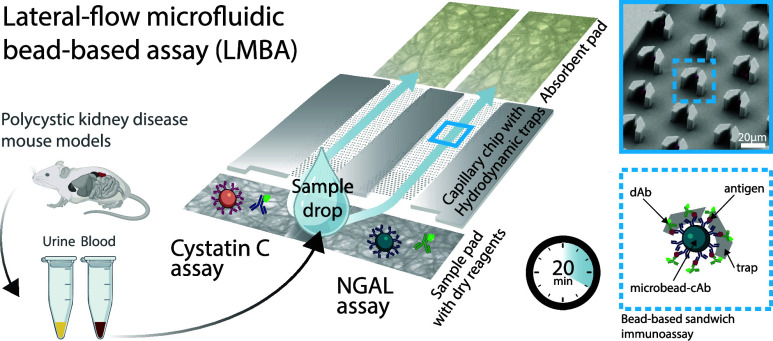

## Introduction

End-stage renal disease (ESRD), the terminal phase of irreversible kidney failure, is a leading cause of death worldwide, and the demand for renal replacement therapies is expected to more than double by 2030.^1^ ESRD can result from acute kidney injury (AKI)^2^ or the progression of chronic kidney disease (CKD).^3^ AKI affects 10–15% of hospitalized patients, with up to 50% incidence in intensive care units, making prompt detection and staging critical to reducing mortality.^2^ CKD impacts an estimated 10% of the global population,^3^ and polycystic kidney disease (PKD) is the most prevalent among hereditary chronic disorders.^4^ Monitoring CKD progression and stratifying patients enables personalized care, delaying dialysis or transplantation.^3^ Given the substantial burden of kidney diseases, deepening our understanding of renal pathophysiology and improving diagnostic tools remains an essential priority. Alongside clinical studies, experimental research relies predominantly on mouse models due to their genetic tractability, physiological similarity to humans, small size, and short gestation.^5^

Established clinical diagnostic criteria for kidney diseases are primarily based on glomerular filtration rate (GFR) estimates from serum creatinine measurements.^2,3,6^ Although creatinine levels are the standard method for staging AKI^2^ and CKD,^3^ they are affected by nonspecific determinants and increase only at advanced kidney damage (>48 h post-AKI onset).^6,7^ Novel biomarkers can improve the assessment of kidney health, facilitating prompt AKI diagnosis, CKD stratification, and correlation with PKD cystic growth.^6^ Cystatin C (CysC) is a filtration marker offering a more accurate GFR estimation than creatinine.^7^ Its levels typically rise within 24 h of AKI^8,9^ and correlate with CKD severity^10^ and PKD progression.^11^ Neutrophil gelatinase-associated lipocalin (NGAL), a glycoprotein secreted by tubular cells during injury, is detectable within 4 h after AKI onset.^7,8,12^ NGAL levels also reflect declining renal function in patients with CKD^13^ and PKD.^14^ By providing complementary insights into kidney injury—filtration dysfunction and tubular damage—the combined detection of CysC and NGAL delivers a more comprehensive and timely diagnosis, enabling earlier identification of AKI and more informative prognosis in CKD and PKD.

Despite increasing clinical relevance, CysC and NGAL quantification is restricted to centralized laboratories and trained personnel, using labor-intensive protocols like enzyme-linked immunosorbent assays (ELISAs) or costly automated immunoanalyzers.^15^ High-throughput systems, such as Luminex, offer multiplexing through bead-based immunoassays, but are not readily deployable outside specialized facilities.^16^ By contrast, lateral-flow immunoassays (LFAs) are widely used for rapid diagnostics, with tests available for both NGAL^17,18^ and CysC.^17^ Although suitable for point-of-care use, LFAs provide only qualitative or semiquantitative outputs, lacking the sensitivity for early detection and patient stratification.^19^ These limitations highlight the need for rapid near-patient quantitative diagnostic platforms that can operate outside centralized analytical laboratories, enabling timely AKI assessment in emergency care and longitudinal CKD monitoring in decentralized clinical settings.^6,15^

Microfluidic systems that implement bead-based assays on miniaturized platforms can yield portable, self-contained diagnostic devices.^15,20,21^ Moreover, this solution facilitates automated workflows with minimal sample volumes and benefits from high surface-to-volume ratios and fast mass transport.^22^ Additionally, beads can be produced in batches with low variability and offer versatility for surface chemical modification and biofunctionalization.^22^ However, existing microfluidic bead-based assays still face limitations in their automation and ease of use. One system has been developed to quantify CysC and a second kidney injury marker (KIM-1), but it involves seven sequential additions of reagents and wash solutions.^23^ Alternative strategies relying on capillary-driven systems using hydrodynamic bead trapping can simplify workflows, yet they often require manual washing,^24^ reagent injections,^25^ or prelabeling steps.^26^ A single-step microfluidic immunoassay using preloaded beads has also been demonstrated.^27^ Nonetheless, these state-of-the-art systems based exclusively on microfluidics face significant design constraints related to material compatibility with microfabrication, capillary force generation, bioreagent storage, and optical detection, ultimately affecting their analytical performance.

We introduce the *lateral-flow microfluidic bead-based assay (LMBA)*, a self-contained, fully automated platform for kidney biomarker quantification (Figure 1). By integrating a microfluidic reaction chamber with independent paper-based components, this hybrid system circumvents the material and design constraints of purely microfluidic devices, offering a versatile and reconfigurable solution. To our knowledge, this is the first system to achieve simultaneous, quantitative detection of CysC and NGAL within 20 min, requiring only sample loading after dilution. The LMBA requires only 1 μL of the original blood or urine sample prior to dilution, making it well-suited for frequent screening and applications where sample volume is highly constrained, such as studies involving very small animals. Leveraging this capability, we employed the system to assess kidney function in two PKD mouse models during early postnatal development. Biomarker levels measured in these models fall within ranges reported in patients with AKI and CKD, supporting the clinical relevance of the assay’s dynamic range. The analytical performance of the LMBA was validated against ELISA, demonstrating its suitability for quantitative biomarker measurements in preclinical samples and highlighting its potential for future translation.

**Figure 1:**
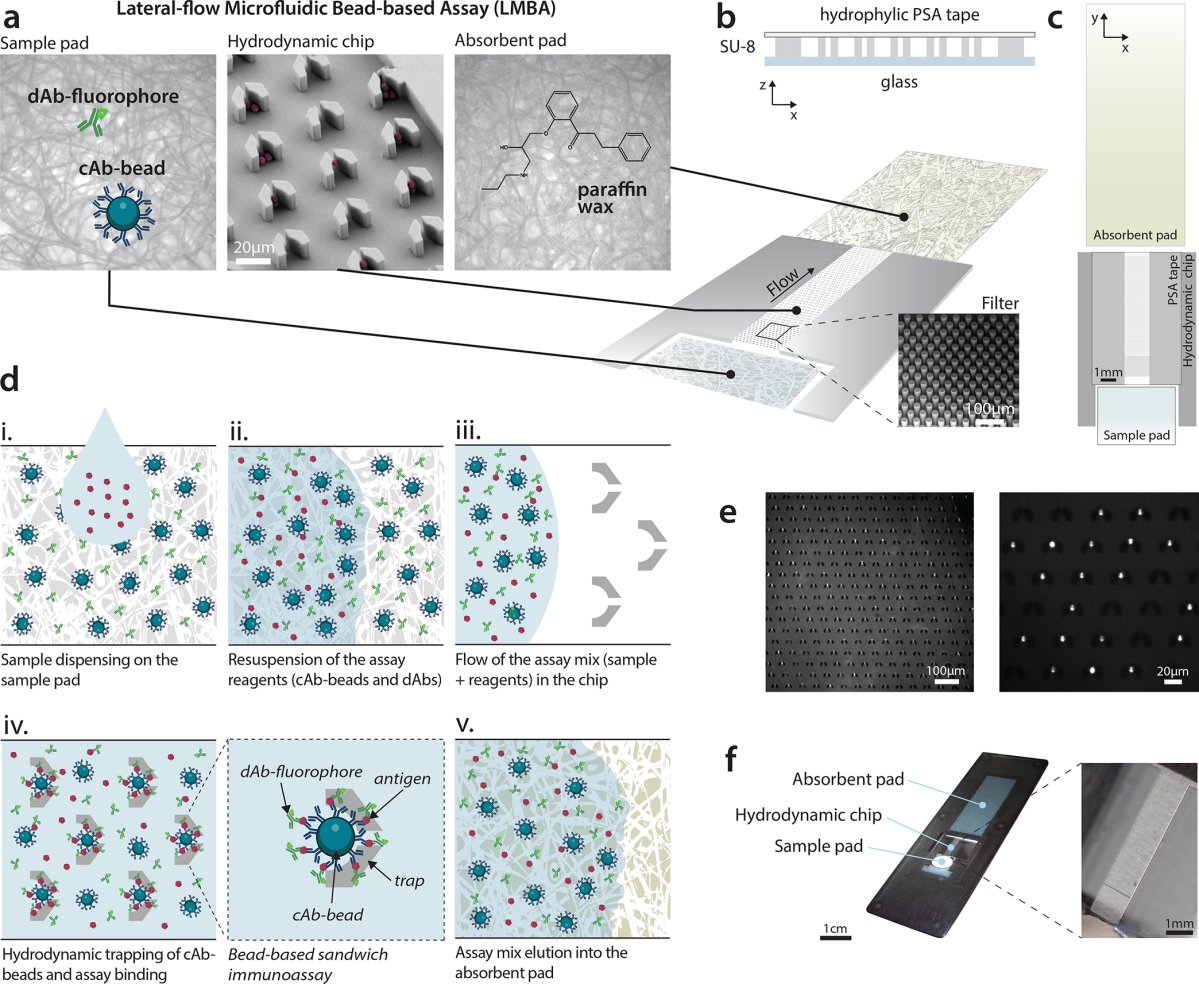
Single-marker lateral-flow microfluidic bead-based assay (LMBA). (a) Schematic representation of the modular LMBA platform comprising three components: the sample pad (cellulose fiber paper, inset) preloaded with immunoassay reagents, specifically, microbeads conjugated with capture antibodies (cAb-beads) and fluorescently labeled detection antibodies (dAbs); the hydrodynamic chip (scanning electron microscopy image, inset) featuring hydrodynamic traps designed to immobilize the cAb-beads against the sample flow; and the absorbent pad (cotton linter paper, inset), which collects the sample and maintains capillary flow via adjusted wettability achieved by paraffin wax functionalization. A filter region (scanning electron microscopy image, inset) is positioned at the hydrodynamic chip entry upstream of the trap array. (b) Schematic cross-sectional (x-z) representation of the hydrodynamic chip layers. The device is fabricated by SU-8 photolithography on a glass wafer substrate and sealed with hydrophilic pressure-sensitive adhesive (PSA) tape. (c) LMBA platform modules depicted to scale (x-y, top view). (d) Working principle of the LMBA: (i) the sample (50 μL total loading volume, corresponding to ∼1 μL of original biological sample under the dilution conditions used in this study) is dispensed on the sample pad, initiating (ii) the resuspension of the reagents (cAb-beads and dAbs); (iii) the assay mix is capillary-driven through the microfluidic chip. (iv) Sandwich immunoassay complexes form on the microbeads retained by the hydrodynamic traps, while (v) the absorbent pad maintains a continuous capillary flow for 20 min. (e) Fluorescence microscopy images of microbeads in the hydrodynamic traps after 20 min of the LMBA binding phase (NGAL, 32 ng/mL in running buffer). (f) LMBA platform modules assembled within a custom-designed cassette, with an inset of the hydrodynamic chip channel. Images taken with Microqubic MRCL700 3D Imager Pro.

## Experimental Section

### Immunoassay Reagents

Commercial ELISA kits were utilized for sandwich ELISA assays targeting NGAL (Bio-Techne, Cat. No. DY1857) and cystatin C (Bio-Techne, Cat. No. DY1238). ELISAs were run according to the manufacturer’s instructions. The antibodies provided with the ELISA kits were also used in bead-based assays in the HBA (homogeneous bead-based assay by bulk mixing in a tube) and LMBA formats. According to the manufacturer’s validation data, these antibody pairs exhibit no significant cross-reactivity with structurally related proteins, except for the partial cross-reactivity of the CysC assay with rat cystatin C reported in the kit documentation. In bead-based assays, the biotin-functionalized antibody in the kit was utilized as the control antibody (cAb) immobilized on streptavidin-coated microbeads. In contrast, the other antibody in the kit served as the detection antibody (dAb) and was linked to the fluorophore. Briefly, 25 μL of 0.5% w/v 7 or 10 μm streptavidin-coated polystyrene beads (Spherotech, Cat. No. SVP-60–5) were centrifuged (5 min at 3000 rpm) to remove the supernatant and then resuspended in an equal volume of 0.1 M Phosphate Buffer (PB; Sigma-Aldrich, Cat. No. P3619). Next, 8 μL of cAbs at 1 mg/mL in 1× PBS (Life Technologies, Cat. No. 10010015) was added to the bead solution and incubated overnight. The cAb-beads were resuspended in 50 μL of 10% Bovine Serum Albumin (BSA; Sigma-Aldrich, Cat. No. A7906) in PBS 1× and incubated for 5 h for blocking. Following the blocking step, the cAb-beads were subjected to three washes with 50 μL of 0.05% Tween 20 (Sigma-Aldrich, Cat. No. P9416) in 0.1 M PB and finally resuspended in 25 μL of 0.1 M PB for long-term storage. The dAbs were conjugated with the Alexa Fluor 647 fluorophores using the Conjugation Kit Lightning-Link (Abcam, Cat. No. ab269823).

Bead-based and ELISA assays were conducted in the same running buffer, consisting of 1% BSA and 0.05% Tween 20 in PBS 1×, and using recombinant NGAL and cystatin C proteins from the ELISA kits for standard dilution calibrators. The effect of buffer composition was assessed by HBA tests, supplementing PBS with 1% BSA and 0.05% Tween 20, as per the running buffer formulation suggested by the ELISA kit provider (Figure S1). This formulation reduced nonspecific interactions with cAb-beads, as evidenced by lower blank signals and variability, resulting in a lower LOD. Additionally, the fluorescence signal at 50 ng/mL NGAL was markedly enhanced in the supplemented running buffer compared to PBS alone.

### Immunoassay Protocols

#### LMBA Assay

After assembling the different modules (sample pad, hydrodynamic chip, and absorbent pad) into the assay cassette, incorporating alignment pins to ensure a stable overlap of the paper component onto the chip, 50 μL of sample was dispensed onto the sample pad. Urine and plasma samples were diluted 50-fold and 80-fold in running buffer, respectively, corresponding to approximately 1 μL of the original biological sample per LMBA assay. The readout was conducted after 20 min. Microbeads captured by the hydrodynamic traps were imaged using brightfield and fluorescence microscopy (Leica DM2500 M equipped with a Zyla 4.2 CMOS camera from Andor Technologies). At least 50 single beads were imaged for each sample condition. Multimarker assays were carried out using the same methodology, with a sample volume dispensed onto the sample pad ranging from 50 to 80 μL.

A custom MATLAB (R2023a, MathWorks) code was developed to quantify fluorescence signals from the microbeads. Figure S2 displays the image processing methodology. First, a flat-field correction was applied to the fluorescence images. Successively, each bead was located in the brightfield images using the MATLAB built-in function “Imfindcircles.” For each bead, the fluorescence signal was computed by averaging pixel intensities within the bead area. Background fluorescence was estimated from a local bead-free region of interest selected adjacent to each hydrodynamic trap using the brightfield image as a spatial reference and subtracted from the bead signal. This local correction compensates for diffuse fluorescence from unbound detection antibodies as well as spatial inhomogeneities across the field of view, including illumination gradients, contaminants, and chip-related optical variations. The background-corrected signal was successively averaged over at least 50 beads for each LMBA assay condition and expressed in arbitrary units (a.u.). Across the NGAL dose–response experiments, the local background signal measured in bead-free regions was 750 ± 150 au, while the standard deviation of the blank-corrected LMBA signal was 3 au.

#### HBA Assay

cAb-beads and dAbs at the same concentrations used in LMBA assays were incubated in vials with turbulent mixing of a 50 μL sample for 20 min, followed by fluorescence microscopy imaging of a 10 μL sample dispensed onto a hemocytometer chamber. The methodology for quantifying fluorescence signals from microbeads in HBA assays was identical to that developed for LMBAs (Figure S2). For interference testing, human serum albumin (Apollo Scientific, Cat. No. BITP1579) and human hemoglobin (Sigma, Cat. No. H7379) were used as representative endogenous interferents for urine and plasma matrices, respectively.

#### ELISA Assay

ELISAs were run according to the manufacturer’s instructions (Bio-Techne, Cat. No. DY1857 and DY1238). ELISA assays were read by an Elx800 microplate reader (BioTek), and the absorbance assay signal at 450 nm was subtracted from the plate background absorbance at 562 nm.

### Dose–Response Curve Analysis

Seven calibrators were measured in duplicate. Blank-corrected data were fitted using a four-parameter logistic regression (4-PL) with inverse-variance weighting. The limit of detection (LOD) and limit of quantification (LOQ) were determined by back-calculating concentrations correspondeding, respectively, to signals three and ten times the standard deviation of the blank. The LOQ establishes the lower limit of the LMBA dynamics. The upper limit is defined as the saturation point, the concentration that yields 95% of the maximum asymptotic response from the 4-PL model. Resolution of molecular concentration (RMC) was calculated from the fitted 4-PL dose–response curve and the propagated uncertainty of the fitted response.^28^ At each analyte concentration *x*_1_, RMC corresponds to the minimum fold-change μ = *x*_2_/*x*_1_ between two concentrations that yields statistically distinguishable assay signals with 95% confidence, based on the standard errors associated with the fitted dose–response curve.

### Hydrodynamic Chip Microfabrication

The hydrodynamic chip featuring hydrodynamic traps was fabricated at the Centre of MicroNanoTechnology (CMi) through SU-8 Kayaku 3025 (Series 3000, Kayaku Advanced Materials; water contact angle 87° ± 2°) photolithography on a glass substrate (4 in. float glass wafer). The mask CAD layout is shown in Figure S3. Before SU-8 photolithography, the glass surface was cleaned with oxygen plasma. The detailed microfabrication protocol is outlined in Table S1. This photolithography protocol yielded a thickness of 26 μm and an approximate resolution of 5 μm. After SU-8 cross-linking, a designed 6 μm gap resulted in fabricated traps measuring 4–5 μm. Postdicing, each hydrodynamic chip was sealed with hydrophilic pressure-sensitive adhesive (PSA) tape (ARflow 93049, Adhesive Research), consisting of a single-coated transparent polyester film with a hydrophilic surface treatment designed for in vitro diagnostic applications requiring enhanced wettability with aqueous biological fluids such as urine and blood. The material was selected because it provides stable capillary wetting, optical transparency for fluorescence imaging, and a thin bonding layer (∼100 μm), minimizing channel distortion while enabling cleanroom-free assembly. The PSA tape was manually cut and positioned on the desired chip area, then gently pressed to ensure proper sealing against the SU-8 channel walls.

Hydrodynamic traps were arranged in rows, with each containing 24 traps evenly distributed across the channel width. The main channel contained 120 rows, with each subsequent row laterally offset by the trap opening size relative to that of the preceding row. Traps accommodated up to two beads vertically and three beads horizontally, but most traps contained a single bead during LMBA assays. Vertical stacking, when observed, yielded higher fluorescence signals compared with single-bead imaging.

### Functionalization of Paper Modules and LMBA System Assembly

The cellulose fiber paper (Merck Millipore, Cat. No. CFSP20300) was cut into 5 mm × 7 mm sample pads. Sample pads were treated with a standard blocking buffer.^29^ The addition of 5% sucrose and 0.05% Tween 20 enhanced bead extraction compared to a 1% BSA blocking buffer. Accelerating the drying process using 80 °C instead of the standard 37 °C^29^ did not compromise blocking efficiency and increased process throughput. Briefly, the sample pads were immersed in the blocking buffer containing 5% sucrose (Sigma-Aldrich, Cat. No. S8501), 1% BSA, and 0.05% Tween 20 in ultrapure water for 1 min, followed by drying at 80 °C for 30 min. After blocking, each sample pad was loaded with 1.5 μL of cAb-beads at a concentration of 0.5% w/v and either 0.7 μL of dAbs at 83 μg/mL for NGAL assays or 0.9 μL of dAbs at 83 μg/mL for CysC assays. Defining a 1× solution with a molar concentration equivalent to 50 ng/mL of CysC in a 50 μL sample, the optimal concentration was 3×, compared to 2× for NGAL. For use in multimarker LMBA platforms, a hydrophobic barrier was created by submerging one edge of the sample pad (1 mm) in molten paraffin wax (Sigma-Aldrich, Cat. No. 327212) at 65 °C, which resolidified and became entangled within the cotton fibers.

Among cotton linter papers commonly used in LFA fabrication, media with high absorption capacity, such as CF5 (Cytiva, CF5 Cat. No. 8115–2250), fully wetted the sample within just 1 min. Consequently, we opted for CF1 (Cytiva, Cat. No. 8111–2250; wicking rate 207 s/4 cm, water absorption 18 mg/cm^2^) for its minimal bed volume. The absorbent cotton linter paper was cut into 11 mm × 30 mm absorbent pads corresponding to a 100 μL bed volume. Absorbent pads were treated with paraffin wax dissolved in toluene, achieving a homogeneous dispersion upon toluene evaporation. Pads were soaked in a solution containing 4 mg/mL of paraffin wax in toluene (Sigma-Aldrich, Cat. No. 32249) for 10 s. Finally, the sample pad with preloaded reagents and the wax-treated absorbent pad were brought into contact with the hydrodynamic chip. These three components were housed in a custom-made cassette to ensure optimal coupling. The cassette was designed in Fusion 360 (Autodesk) and fabricated on a J55 Prime Full Color 3D Printer (Stratasys) in VeroClear material.

### Mouse Lines and Collection of Urine and Plasma Samples

All animal experiments were approved by the Veterinary Office of the Swiss canton of Vaud. The derivation of *Bicc1* ^–/–^ mice and their genotyping by PCR have been previously described.^30^ Here, *Bicc1* ^+/−^ heterozygous females on a C57BL/6J inbred background were outcrossed to BALB/c males for one generation. Intercrossing of heterozygous F1 offspring served to obtain *Bicc1* ^–/–^ homozygotes with increased hybrid vigor that survived for at least 2 weeks after birth, in contrast to C57BL/6J inbreds that rarely survived longer than 3 days. WT controls were littermates. *Bicc1* cKO-Ksp mice were generated by introducing the Ksp1.3/Cre transgene (JAX stock #012237)^31^ in *Bicc1* ^lox/lox^ mice that will be described elsewhere in more detail. Briefly, exon 4 of *Bicc1* was flanked with LoxP sites using easy-CRISPR-assisted homologous recombination in C57BL/6J zygotes.^32^ PCR genotyping used the following primers: bicc1ex4_F1 5′-TTCGCAGTGGTGTGATTTGC-3′; bicc1ex4_R1 5′-TGTAGCACTAACAAGGGCCA-3′; bicc1ex4_F2 5′-CAACACATTCCCCAGACCTT-3′; and bicc1ex4_R2 5′-GGGCTCCTATGACCTCAGAA-3′. *Bicc1* mutants and their corresponding WT littermates were decapitated at postnatal days P5, P7, P9, P11, or P14. Blood and urine were collected at the time of sacrifice. Blood samples were centrifuged in heparin-coated tubes to separate the plasma. The plasma was subsequently collected and snap-frozen in liquid nitrogen. Urine was obtained directly from the bladder and snap-frozen in liquid nitrogen. Samples were stored at −80 °C before testing. Samples were diluted in running buffer at 1:50 and 1:80 for LMBA tests of urine and plasma, respectively, and at 1:500 and 1:800 for ELISAs.

### Statistical Analysis

Unless otherwise stated, results are displayed as mean ± SD by Origin 2022 (OriginLab Corporation). To compare two data sets, a two-tailed Student’s *t* test for independent samples was used to assess statistical significance after the Shapiro–Wilk normality test. One-way analysis of variance (ANOVA), followed by Tukey’s test for multiple comparisons, was used to assess the statistical significance of results across more than two data sets. Dose–response curves were fitted using a four-parameter logistic model with inverse-variance weighting.

## Results and Discussion

### LMBA Workflow and System Architecture

The LMBA system comprises three modules through which the sample progresses sequentially: the sample pad, the hydrodynamic chip, and the absorbent pad (Figure 1a–c). A single-step protocol (Figure 1d), typical of lateral-flow assays, was devised to achieve quantitative bead-based assays by integrating microfluidics with paper modules. The device features a self-contained, fully autonomous process for antigen quantification using sandwich immunoassays.

The sample pad is preloaded with microbeads decorated with capture antibodies (cAb-beads) and detection antibodies (dAbs) in a dry form, which are released into the solution upon sample dispensing. Within seconds, the sample and resuspended reagents are drawn into the hydrodynamic chip via capillary action. The detection region features hydrodynamic traps that retain the cAb-beads against the flow sustained by the absorbent pad, allowing for a 20 min binding phase under convection. Sandwich assay complexes form on the trapped cAb-beads, and the resulting signal is quantified by fluorescence (Figure 1e and Video V1). The beads are released from the traps when the flow stops, providing a control to verify the continuous sample convection.

This modular approach offers exceptional design flexibility. The sample pad collects the entire sample volume in a small footprint and serves as a reservoir for the duration of the assay binding in the flow. Additionally, it can undergo dedicated treatments to optimize reagent storage and resuspension. While assay binding and readout are controlled by the hydrodynamic chip design, flow rate and total assay time are exclusively determined by the absorbent pad. Moreover, the LMBA is easily parallelizable for multimarker testing. Sample pads can be compartmentalized to store separate reagents, and absorbent pads can set different flow rates, ensuring the independent operation of simultaneous assays and avoiding reciprocal constraints. A 3D-printed cassette was developed to ensure reproducible pairing of the LMBA components (Figure 1f and Video V2).

Although fluorescence microscopy was used in this study for analytical validation under controlled imaging conditions, Video V2 demonstrates that the 7–10 μm microbeads remain spatially resolvable with a compact optical instrument (MRCL700 3D Imager Pro, Microqubic AG) that employs simpler, lower-cost optics than a conventional laboratory microscope. Once this bead-resolving magnification is achieved, fluorescence integration requires only standard optoelectronic components, such as LED excitation and excitation/emission filtering, as already implemented in portable fluorescence immunoassay readers.

### LMBA Dose–Response for NGAL Quantification and Comparison to ELISA

We characterized the LMBA dose–response for NGAL, an early-stage predictor of disease progression in PKD.^14^ Seven calibrators were measured in duplicate (Figure 2a), following standard practice for quantitative ligand-binding assays.^33,34^ Data were fitted using a four-parameter logistic regression (4-PL), yielding an R-squared value of 0.99. The LMBA limit of detection (LOD) is 220 pg/mL, and the limit of quantification (LOQ) is 360 pg/mL, while the saturation point is 400 ng/mL, corresponding to a dynamic range of 3 orders of magnitude.

**Figure 2:**
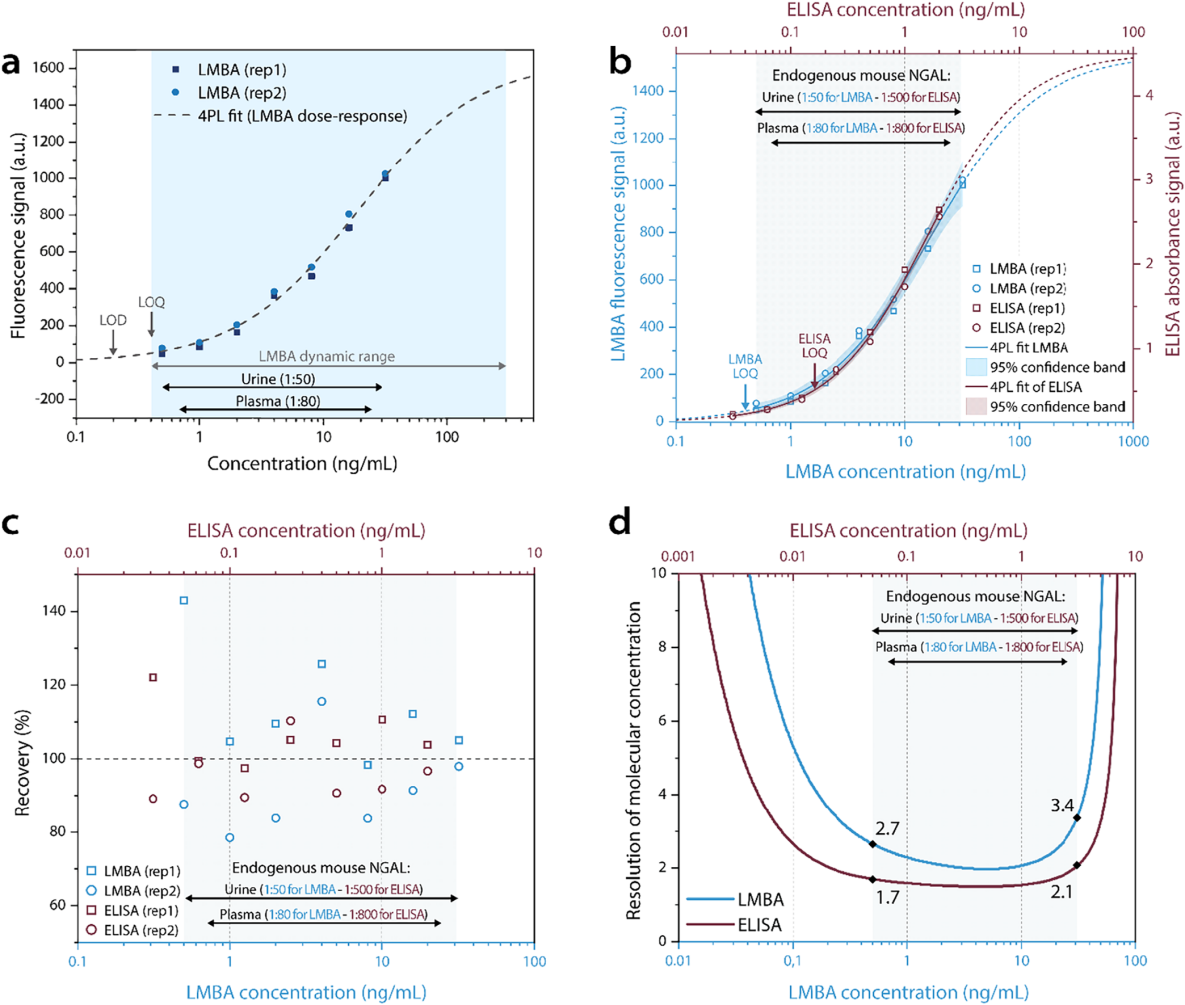
LMBA dose–response and performance comparison with ELISA. (a) LMBA dose–response curve for neutrophil gelatinase-associated lipocalin (NGAL) obtained from two replicates (rep 1,2) of standard dilution calibrators in running buffer (1% BSA and 0.05% Tween 20 in PBS 1×). Measured fluorescence signals were blank-corrected (*n* = 5 blank measurements) and fitted using a four-parameter logistic (4-PL) regression model, yielding an R-squared value of 0.99 (dashed line). The limit of detection (LOD) and limit of quantification (LOQ) were determined as the concentrations corresponding to three and ten times the standard deviation of the blank, respectively (*n* = 5). The LMBA dynamic range (light blue area) is compared with the endogenous NGAL ranges in urine and plasma samples from the mouse cohort under study, with 50-fold and 80-fold dilutions in running buffer, respectively. (b) LMBA dose–response curve (same data set as panel (a); blue axes) superimposed on the ELISA dose–response curve (*n* = 2 replicates of calibrators; red axes). Both data sets were fitted with a 4-PL model, and each was plotted on an *x*-scale centered at the inflection point (EC50) and a *y*-scale between the 4-PL asymptotic values. Both assays utilized the same antibody pair and recombinant NGAL protein in the running buffer. Endogenous NGAL ranges in mouse samples (gray area) are rescaled by their dilution factors: urine diluted 50-fold for LMBA and 500-fold for ELISA, plasma diluted 80-fold for LMBA and 800-fold for ELISA. (c) Accuracy profiles illustrating the recovery (%) of each calibrator point of panel (b) for LMBA (blue) and ELISA (red). Recovery was calculated as the ratio (%) of the back-calculated concentration to the nominal calibrator concentration. (d) Resolution of molecular concentration (RMC) curves for LMBA (blue) and ELISA (red); the intersections of the gray area indicate RMC values with 95% confidence for the NGAL concentration boundaries within the endogenous concentration range of the mouse sample cohort under study.

The LMBA was calibrated in running buffer following the formulation recommended by the ELISA kit provider. The NGAL LMBA antibody pair was sourced from the same commercial kit, allowing a direct comparison between the two assays (Figure 2b). Each dose–response curve was centered on the *x*-axis at its inflection point (EC50, the concentration at which the response reaches 50% of the dynamic range^35^) and was normalized between its fitted asymptotes. LMBA and ELISA exhibit comparable steepness of the 4-PL dose–response curves, covering 3 orders of magnitude in concentration. The ELISA dose–response is shifted by 1 order of magnitude toward lower concentrations, with the EC50 at 1.6 ng/mL for ELISA and 17 ng/mL for LMBA, thereby allowing ten times larger dilution factors than those used in LMBA. However, the ELISA LOD and LOQ are 110 pg/mL and 170 pg/mL, respectively, only 2-fold lower than those of LMBA, and the ELISA saturation point occurs at 25 ng/mL. Overall, the LMBA offers similar sensitivity to that of ELISA, but its dynamic range (360 pg/mL–400 ng/mL) outperforms that of ELISA (170 pg/mL–25 ng/mL). While ELISA benefits from enzymatic amplification, shifting its dose–response by one decade toward lower concentrations, it is affected by blank variability, resulting in an LOQ comparable to that of LMBA.

Accuracy profiles for LMBA and ELISA were quantified by recovery rates (Figure 2c). The LMBA 4-PL curve exhibits high accuracy, with a mean recovery of 87% ± 11%. ELISA calibrators tested within the same microwell plate showed a similar recovery of 91% ± 8%, consistent with the manufacturer’s validation (93%). Dose–response curves were further evaluated using the resolution of molecular concentration (RMC), a recently introduced metric that quantifies the significant changes in concentration discernible by an assay.^28^ Ultimately, the assay accuracy evaluated through recovery factors correlates with fitting quality and thus with the resolution of molecular concentration. We assessed the RMC with 95% confidence (Figure 2d). Over the endogenous NGAL levels in mouse samples (Tables S2 and S3), the LMBA demonstrates an RMC capable of resolving approximately 3-fold concentration changes (RMC ∼ 3), compared to 2-fold changes for ELISA (RMC ∼ 2). LMBA is a single-use system that might be affected by variability in dry-form reagent performance, module functionalization, and assembly. The superior accuracy of ELISA likely stems from replicate testing within the same microwell plate, reducing inconsistencies.

Homogeneous assays (HBAs) performed by bulk-mixing assay reagents were used to evaluate the assay response in diluted mouse samples (Figure S4). We identified dilution factors—50-fold for urine and 80-fold for plasma—that suppress matrix effects while preserving the assay’s analytical performance. Consequently, we considered the LMBA dose–response curve in running buffer as the calibration for quantifying samples of different origins. This has the additional benefit of obviating the need to deplete endogenous analytes from calibration matrices through labor-intensive and costly purification processes.^36^

In addition to evaluating the NGAL target quantification performance and assessing matrix effects, we performed interference testing focusing on key endogenous components commonly present in urine and plasma samples. For urine kidney-marker immunoassays, elevated albumin levels (200 mg/L) associated with macroalbuminuria represent a major potential interferent.^37^ For plasma assays, free hemoglobin (300 mg/dL) arising from hemolysis is a well-recognized source of analytical interference^38^ and is particularly relevant in this study as blood collection from mice before postnatal day 14 was technically challenging. Accordingly, approximately 20% of collected plasma samples exhibited visible hemolysis, as indicated by a red coloration. To reproduce the effective interfering concentrations under the assay conditions, these pathological concentrations were divided by the sample dilution factors used in this study (50-fold for urine and 80-fold for plasma). We therefore evaluated assay robustness by measuring NGAL EC50 output signals via HBAs in the presence of 4 mg/L albumin and 3.75 mg/dL hemoglobin (Figure S5), observing limited interference levels of 6% and 9%, respectively. These interference levels remained below the 10% threshold commonly accepted in CLSI interference guidelines,^35^ supporting assay specificity under biologically relevant conditions. The results are consistent with the matrix-effect experiments (Figure S4), in which recovery values remained above 90% at the selected dilution factors despite the presence of visibly hemolyzed plasma samples, supporting the robustness of the assay under biologically relevant interference conditions.

### Integrated Design of Reagent Storage, Bead Trapping, and Capillary Flow

The system components were engineered for an LMBA assay using a 50 μL sample volume. A cellulose fiber sample pad, commonly employed in LFAs, stores the reagents in a dry form and is sized to absorb the volume of the loaded sample. Nonspecific adsorption of preloaded reagents was mitigated by pretreating the sample pad with a blocking buffer (Figure S6). Moreover, storage of sample pads containing dry reagents (cAb-beads and dAbs) at room temperature in a sealed pouch with a silica gel desiccant resulted in only a 9% average decrease in signal after 28 days, compared with LMBA measurements performed using freshly prepared reagents in liquid form (Figure S7). The number of preloaded beads (approximately 4 × 10^4^) was adjusted to ensure robust occupancy of the 2640 hydrodynamic traps despite bead losses during resuspension and flow, consistently enabling fluorescence quantification from at least 100 trapped beads per assay. Across three independent devices, the trap occupancy after the 20 min LMBA was 78 ± 5%.

HBA assays, in which binding is performed in bulk by mixing cAb-beads with dAbs in the sample, were used to determine the blocking strategy and the reagent concentrations to meet the required dynamic range and quantification performance (Figures S6 and S8). Eliminating wash steps is compelling for a fully automated protocol but imposes a trade-off between the assay signal and background fluorescence. For this reason, the LMBA response is particularly sensitive to dAb concentrations, which were carefully optimized (Figure S8a) to align with endogenous NGAL levels in mice, scaled according to the chosen dilution factors (Tables S2 and S3).

The hydrodynamic chip was designed to retain beads as small as 7 μm, leveraging the principle introduced by Di Carlo et al.^39^ Each hydrodynamic trap features a gap spanning the channel height, a previously proposed approach.^40–42^ This geometry enables bead trapping across the entire channel height, mitigating mass-transport limitations. The chip includes an upstream opening to accommodate the sample pad and a filtering region (25 μm gap size) at the channel entrance. This filtering region was sufficient under the diluted-sample conditions used in the current analytical validation, and no clogging of the hydrodynamic bead traps was observed during the 20 min assay. Future implementations at lower dilution factors or with complex clinical matrices may incorporate additional sample-conditioning membranes upstream of the sample pad to remove cells, debris, or protein aggregates. Capillary pressure generation requires at least two channel walls with contact angles below 60°.^43^ As SU-8 surfaces are hydrophobic, a pressure-sensitive adhesive (PSA) tape with a hydrophilic coating is selected for sealing due to its ease of application, reproducibility, optical transparency, and cleanroom-free bonding.

The composition and wettability of the downstream absorbent pad material regulate the sample flow, thereby dictating assay binding kinetics and time-to-equilibrium, strongly influencing the LMBA readout signal. Wicking rate measurements with a 50 μL running buffer were conducted by tracking the flow front (Figure 3a and Video V3). Capillary flow front advancement decreased proportionally to the square root of time, as is typical in porous media.^44^ Native paper exhibited an initial rapid rate of approximately 20 μL/min, diminishing to 6 μL/min after 7 min, beyond which the flow front became indiscernible. Such an initial high-flow phase immediately after resuspension causes rapid elution of dAbs at the sample flow front. Additionally, for a finite volume, a slower flow allows more time for assay binding.^45^ We thus functionalized the paper with wax to reduce its wettability.^46^ Wax concentrations of 2, 3, 4, and 5 mg/mL were tested. The paper became impermeable at 5 mg/mL, whereas 4 mg/mL yielded the most suitable flow characteristics (Figure S9), maintaining flow rates between 2 and 4 μL/min with improved temporal stability during the first 10 min of capillary flow (coefficient of variation, CV = 7–15%) compared with untreated pads (CV = 31–42%). Treated pads maintained the capillary flow for 20–25 min, as confirmed by continuous hydrodynamic bead immobilization, despite the flow becoming optically indiscernible in the paper after 10 min. To assess the reproducibility of the manual wax-functionalization process, flow rates were compared at 5 min for independently fabricated absorbent pads prepared from different paraffin wax/toluene baths. The intrabatch CV was 5–6%, while the interbatch CV was 17% (Figure S9). Despite batch-to-batch variation in flow rate, LMBAs assembled with absorbent pads from independent batches still yielded a high recovery of NGAL calibrators (87 ± 11%, Figure 2c), indicating that the observed variability did not compromise assay quantification performance.

**Figure 3:**
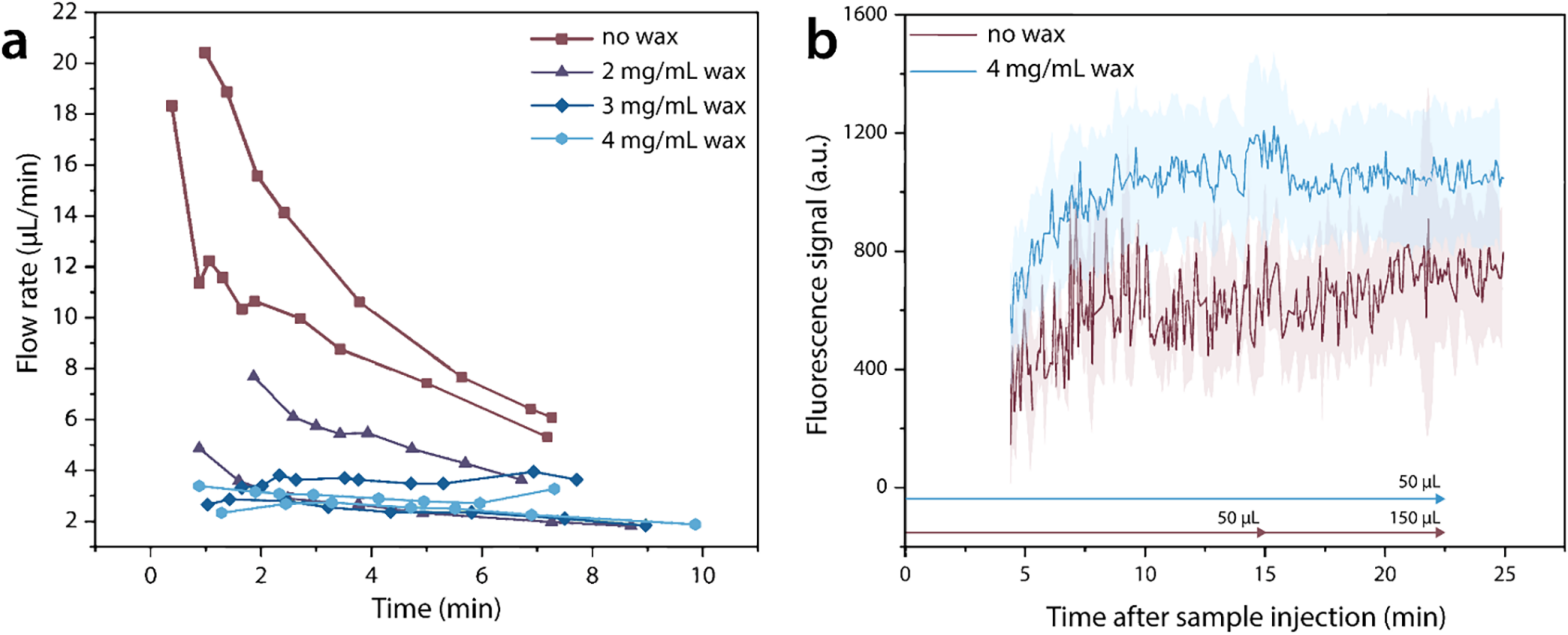
Absorbent pad wettability and LMBA binding kinetics. (a) Time-dependent measurements of capillary flow rate across absorbent pads functionalized with varying concentrations of paraffin wax. Each condition was tested in two independent experiments. (b) Comparative analysis of assays using untreated absorbent pads (requiring 150 μL of sample) versus pads treated with 4 mg/mL paraffin wax (requiring 50 μL). The fluorescence signal was recorded during the LMBA binding phase with a 16 ng/mL NGAL solution. Solid lines represent the mean fluorescence across three independent experiments per condition (10 beads per experiment), with shaded areas indicating the standard deviation. Time *t* = 0 corresponds to the sample dispensing onto the sample pad; the mean fluorescence signal was measured after at least 10 beads were trapped within the same field of view.

To evaluate binding kinetics under different convection conditions (untreated versus wax-treated pads), we monitored the LMBA fluorescence during the flow of an NGAL solution at the LMBA EC50 (Figure 3b). While the wax-treated absorbent pad facilitated continuous sample flow over 25 min using only 50 μL of sample, the untreated pad required dispensing a total of 150 μL. Untreated pads yielded lower fluorescence intensities, as confirmed by end point fluorescence measurements (Figure S10). We observed a 33% increase in signal when using wax-treated absorbent pads compared to untreated ones. The assay time was therefore set to 20 min to ensure readout at equilibrium.

Unlike capillary pumps integrated into microfluidics,^27^ employing an independent absorbent pad circumvents constraints imposed by chip wettability, microfabrication, and capillary pump footprint. The design flexibility offered by the absorbent paper pad allows for precise flow rate control, thereby optimizing the analytical performance. Moreover, modifying the paper wettability enables finer tuning of the flow rate than relying on pore size selection from the limited variety commercially available. Additionally, the LMBA closely mirrored the HBA dose–response curve (Figure S8b), indicating that mass transport through capillary-driven convection is as efficient as turbulent vial mixing.

### Quantification of NGAL Levels in PKD Mouse Models

Polycystic kidney disease leads to renal failure through the formation of fluid-filled cysts, typically due to autosomal dominant mutations in polycystin-1 or polycystin-2 or recessive mutations in fibrocystin.^47^ Genetically engineered mouse models have been developed to investigate cyst formation,^5,47^ including a PKD phenotype obtained through inactivation of the *Bicc1* gene encoding the BicC Family RNA Binding Protein 1 (BicC1).^48^ In human BicC1, only heterozygous mutations have been reported in two pediatric cases of renal cystic dysplasia, consistent with the gene’s essential functions during development.^49^ Studies in mice established that Bicc1 acts both upstream and downstream of polycystin-1 and polycystin-2.^48^ However, the precise mechanisms of cyst development in this and orthologous mouse models of human PKD remain incompletely understood.^50^

Homozygous mutant *Bicc1* ^–/–^ mice show 50% mortality in utero and die shortly after birth.^30^ To overcome this limitation, we used Ksp-Cre^31^ to generate a conditional knockout lacking BicC1 specifically in distal tubules and collecting ducts (cKO-Ksp) (Figure 4a). The single-marker LMBA dose–response for NGAL (Figure 2) was generated in running buffer after confirming appropriate dilutions to neglect matrix effects (Figure S4). This single calibration curve is suited for LMBA quantifications of NGAL in both diluted urine and plasma samples. NGAL was quantified by LMBA and ELISA in urine and plasma samples from *Bicc1* mutants and their wild-type (WT) littermates during the second half of the first and second weeks after birth (Figures 4b,c and S11). Due to the small size of newborn mice, available sample volumes were limited, typically to only a few microliters. Notably, our LMBA system required only 1 μL of the original sample prior to dilution, making it particularly suitable for this study.

**Figure 4:**
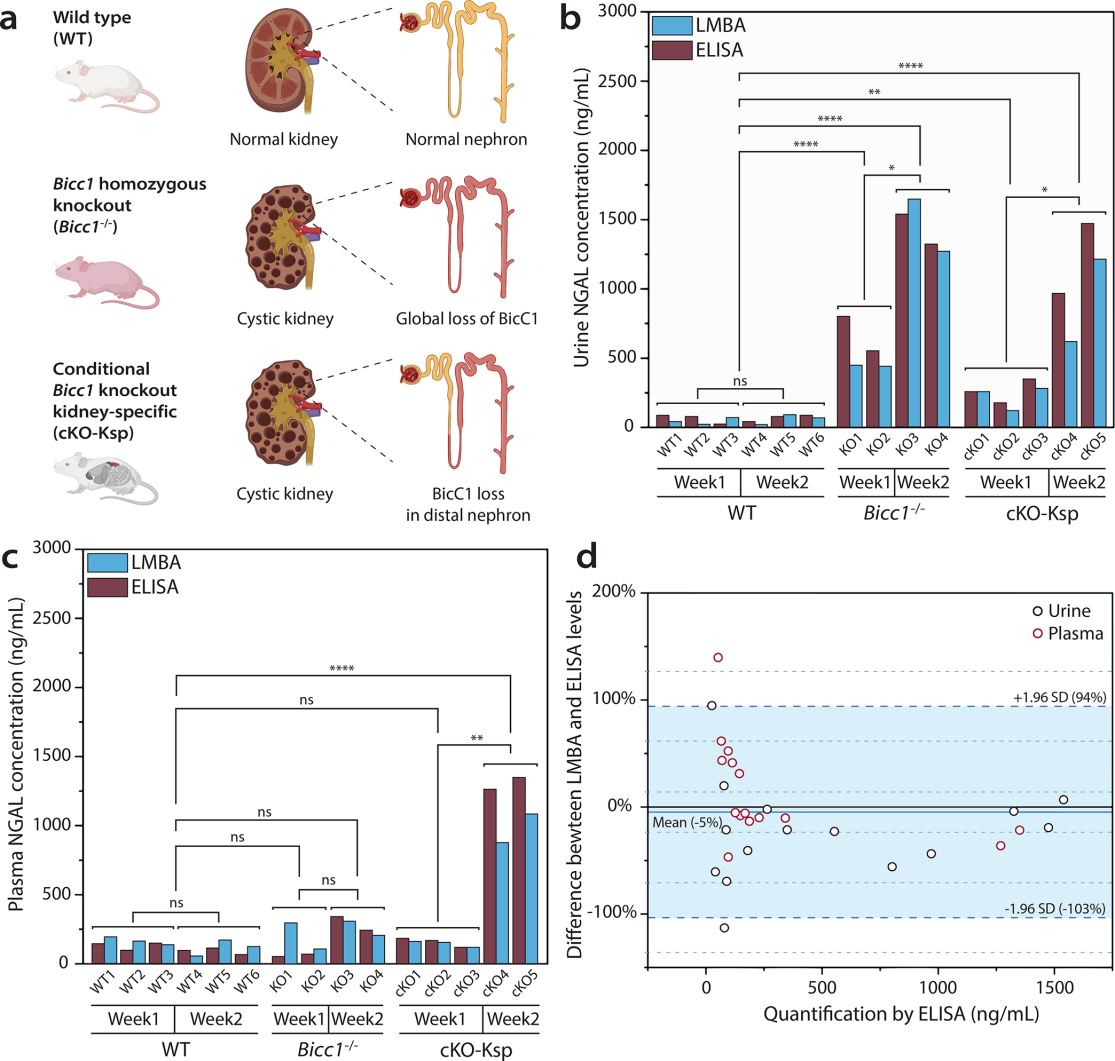
Quantification of NGAL in mouse samples. (a) Schematic representation of the two cystic kidney models examined, including *Bicc1* ^–/–^ mice, characterized by a global loss of BicC1, and cKO-Ksp mice, where Ksp-Cre deletes BicC1 specifically in distal nephron segments that are beyond proximal tubules. (b) Urine from mice of the indicated *Bicc1* genotypes and their WT littermates, and (c) plasma samples from the same animals were quantified using LMBA (light blue) and ELISA (red). Samples were analyzed between days 5–7 (week 1) or between days 10–14 (week 2). Statistical significance was determined via one-way analysis of variance (ANOVA) followed by Tukey’s test for multiple comparisons, after assessing normality with the Shapiro–Wilk test (*p ≤ 0.05, **p ≤ 0.01, ***p ≤ 0.001, ****p ≤ 0.0001). Reported concentrations correspond to NGAL in the original undiluted sample. (d) Bland–Altman correlation analysis comparing LMBA and ELISA results for the data sets shown in panels (a) and (b). The *x*-axis represents ELISA quantifications (reference method), while the *y*-axis shows the percentage difference between LMBA and ELISA values. The mean difference (solid line) and 95% limits of agreement (mean ± 1.96 SD; thick dashed lines) are shown, with their respective 95% confidence intervals indicated as thin dashed lines.

Already during the first week after birth, urinary NGAL (uNGAL) levels were elevated in both *Bicc1* ^–/–^ and cKO-Ksp compared to WT mice. *Bicc1* ^–/–^ mice displayed higher uNGAL levels than cKO-Ksp mice, correlating with the presence of fluid-filled cysts in both proximal tubules and distal nephron segments due to the global loss of Bicc1.^51^ Urinary NGAL levels also rose above the WT baseline upon conditional ablation of *Bicc1* by Ksp-Cre in cKO-Ksp animals, albeit less severely than in *Bicc1* ^–/–^ mice, consistent with the fact that Ksp-Cre activity is absent in proximal tubules and limited to more distal nephron segments.^31^ uNGAL levels further increased in both models during the second week, reflecting a rise in cyst number and size. A critical function of Bicc1 is to curb the levels of cAMP production by adenylate cyclase 6,^51^ a known driver of cystic growth in PKD patients carrying mutations in polycystin-1 or -2.^52,53^ However, a floxed allele to profile PKD-like features in this nonorthologous disease model has been lacking. Here, we found that both *Bicc1* ^–/–^ and cKO-Ksp mutant mice resemble PKD patients also in upregulating uNGAL,^13,54^ thus extending the validity of uNGAL as a chronic kidney disease marker to these mouse models.

Plasma NGAL (pNGAL) exhibited a less pronounced increase compared to uNGAL. Elevated pNGAL levels were detected solely in cKO-Ksp mice during the second postnatal week, whereas *Bicc1* ^–/–^ mice did not show such an increase despite presenting a more severe phenotype. Earlier sample collection (P10 of *Bicc1* ^–/–^ vs P14 of cKO-Ksp) might explain the discrepancy, as pNGAL levels typically rise only at advanced tubular injury. Moreover, *Bicc1* ^–/–^ samples exhibited strong hemolysis, which may have interfered with the binding assay. Higher uNGAL levels than those of pNGAL are due to local production of this marker by damaged kidney tubules, leading to its direct release in urine. Indeed, uNGAL is considered a more effective marker of early kidney injury than pNGAL in PKD^13^ and AKI.^8^

Remarkably, even the smallest significant NGAL spike in this cohort—a 4-fold increase detected in cKO-Ksp urine samples in the first postnatal week vs WT—was greater than the minimum detectable increase for LMBA, corresponding to a 3-fold (RMC ∼ 3). Across 30 urine and plasma samples, LMBA quantifications closely matched those obtained using ELISA, with a mean recovery of 72 ± 20%. Both methods yielded the same significance ranges (*p*-values) for NGAL spikes relative to basal levels in this cohort. Linear regression analysis also showed a strong correlation, with an *R* ^2^ value of 0.93 and a Pearson correlation coefficient of *r* = 0.97 (Figure S12). A Bland–Altman analysis (Figure 4d) revealed that differences between the LMBA and ELISA quantifications ranged from 60% to 100% for NGAL values corresponding to basal levels in WT mice (25–135 ng/mL). Notably, for NGAL spikes above the WT baseline—such as those observed in PKD urine (180–1540 ng/mL) and plasma during the second week (230–1950 ng/mL)—differences systematically remained below 50%, confirming the LMBA’s effectiveness in detecting elevations indicative of PKD.

Precision analysis over independent measurements of spiked and endogenous NGAL samples (Figure S13) revealed an interassay CV of 9% ± 6% across different LMBA single-use devices, meeting the acceptable threshold for immunoassay validation (CV < 20%).^55^ ELISA conducted in different microwells of the same plate exhibited a similar CV of 7% ± 2% (intra-assay). Table 1 summarizes the assay performance of LMBA and ELISA. Notably, the LMBA’s analytical performance for NGAL (Table 1) is compatible with clinical quantification requirements in human samples, as NGAL levels typically rise at least 3-fold in urine and plasma of PKD patients,^13,54^ and within 4 h of AKI onset.^9^ All NGAL calibrators (Figure 2a) and mouse-sample measurements (Figure 4b,c) were performed using dry-stored sample pads from the same functionalization batch. The measurements were conducted approximately 50 days apart, during which the LMBA maintained strong agreement with ELISA and low interassay CVs, further supporting reagent storage stability.

**Table 1:** Analytical Performance of LMBA and ELISA for NGAL Quantification^a^

	LMBA	ELISA
limit of detection, LOD (pg/mL)	220	110
limit of quantification, LOQ (pg/mL)	360	170
saturation point (ng/mL)	400	25
dynamic range (ng/mL)	10^–1^–10^2^	10^–1^–10
resolution of molecular concentration, RMC	2.7–3.4	1.7–2.1
accuracy—recovery (%)	87 ± 11	91 ± 8
precision—coefficient of variation, CV (%)	9 ± 6	7 ± 2

a The assay limit of detection (LOD) and limit of quantification (LOQ) were determined as the concentrations (ng/mL) corresponding to three or ten times the standard deviation of the blank measurements (n = 5), respectively. The saturation point is the concentration that yields 95% of the maximum asymptotic response in the four-parameter logistic (4-PL) model. The dynamic range, expressed in orders of magnitude, is the concentration range between the LOQ and the saturation point. Resolution of molecular concentration (RMC) at 95% confidence is calculated for the boundaries of the endogenous concentration range in the mouse sample cohort. Accuracy was evaluated using recovery (mean ± standard deviation) of quantified calibrators relative to their nominal concentrations; we measured seven calibrators in duplicate and back-calculated using a 4-PL fit of the dose–response curve. Precision was assessed through the coefficient of variation (CV; mean ± standard deviation), computed as the ratio of the standard deviation to the mean across multiple independent measurements.

### Multimarker LMBA Detection of NGAL and CysC

Having established the LMBA platform as an analytically optimized assay for NGAL detection, we next extended it to a double-marker configuration by integrating cystatin C as a secondary target to demonstrate the feasibility of a single-step, spatially multiplexed workflow (Figure 5a–c). The fully automated 20 min protocol remained unchanged, with the two assays run separately through spatial multiplexing. Two single-marker modules were parallelized, maintaining a fully automated procedure requiring only a single sample dispensing. The simultaneous assays took place in two separate regions to avoid cross-contamination (Video V4). The different assay reagents were loaded onto distinct compartments of the sample pad, separated by a hydrophobic wax barrier. When the sample was dispensed, wicking both compartments, the barrier prevented cross-mixing of the reagents before they entered the two separate channels on the hydrodynamic chip. In the immunoassay experiments reported here, the sample was distributed across both compartments using a pipet tip to ensure homogeneous wetting for assay validation. Nevertheless, proof-of-concept experiments demonstrated that a single droplet dispensed at the center of the dual-pad interface using a disposable transfer pipet reproducibly initiated capillary flow in both channels.

**Figure 5:**
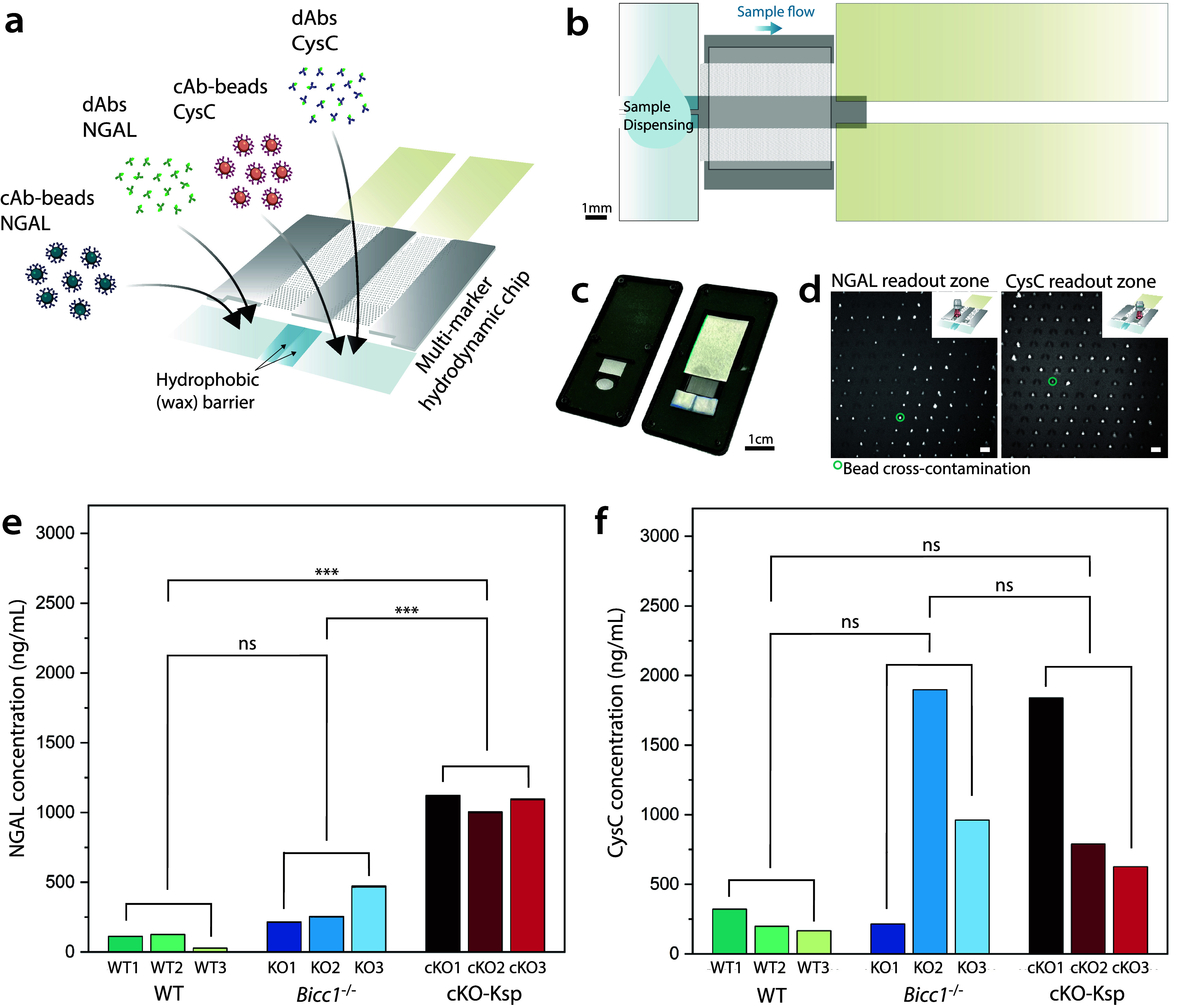
Multimarker LMBA platform and quantification of mouse plasma samples. (a) Schematic representation of the modular LMBA architecture, enabling fully automated and concurrent quantification of NGAL and CysC. The platform features a dual-compartment sample pad with preloaded assay-specific reagents (cAb-beads and dAbs) separated by a hydrophobic barrier, a hydrodynamic chip with two parallel microchannels containing hydrodynamic traps, and two independent absorbent pads for sample collection. (b) Top-view illustration of the LMBA platform components depicted to scale. (c) Photographic image of the fully assembled system within the custom-designed cassette. A single absorbent pad was utilized for this implementation. (d) Representative fluorescence microscopy images of the two parallel microchannels, showing trapped beads for NGAL quantification (7 μm; left) and CysC quantification (10 μm; right). Instances of bead cross-contamination between microchannels are highlighted with circles. Scale bar = 20 μm. Simultaneous quantification of (e) NGAL and (f) CysC in plasma from the indicated *Bicc1* mutants and WT littermates of cKO-Ksp mice. Samples were diluted 1:80 in running buffer. Plasma samples were collected during the second postnatal week (day P10 for *Bicc1* ^–/–^ and day P14 for cKO-Ksp). Statistical significance was determined via one-way analysis of variance (ANOVA) followed by Tukey’s test for multiple comparisons, after assessing normality with the Shapiro–Wilk test (*p ≤ 0.05, **p ≤ 0.01, ***p ≤ 0.001). Reported concentrations correspond to biomarker levels in the original undiluted sample.

In the present study, we aimed at validating the operation of the dual-marker automated workflow, and the flow in both channels was set through a single absorbent pad with wettability optimized for NGAL. Alternatively, using two absorbent pads with different wettabilities can effectively set two independent flow rates on the parallel channels. The spatial separation of the two assays allowed for fluorescence readouts using the same fluorophore. Similar to the single-marker assay, resuspended beads were blocked by the 5 μm hydrodynamic traps against the sample flow for 20 min. To differentiate between the assays, 10 μm beads were used for the CysC assays and 7 μm beads for the NGAL assay. Cross-contamination was detected by image processing (Figure 5d).

As we opted for a single sample dispensing, the dynamic range of the CysC LMBA was set to match the dilution factors established for NGAL. The CysC dose–response curve (Figure S14) showed an LOD and LOQ of 100 and 200 pg/mL, respectively, consistent with the NGAL assay performance. Overall, the CysC LMBA dynamic range (0.2–520 ng/mL) broadly accommodated the levels of the mouse sample cohort. The CysC assay yielded a recovery rate of 72% ± 18% for calibrators, compared to 87% ± 11% for NGAL.

The higher variability observed for CysC reflects the use of assay conditions optimized for NGAL rather than for CysC-specific binding kinetics, including flow rate and reagent concentrations. As a result, the CysC assay in this configuration does not achieve the same level of analytical performance as NGAL. Despite the larger variability, the obtained resolution of the CysC assay (RMC ∼ 4) was sufficient to detect biomarker elevations in the PKD mouse models investigated in this study, in which CysC increased more than 4-fold above the WT basal level, as confirmed by ELISA. Considering reported clinical dynamics, where CysC levels typically increase at least 3-fold in CKDs^56^ or 2-fold in AKI,^8^ the current CysC LMBA does not yet achieve the analytical resolution required to robustly quantify such changes. In contrast, the NGAL assay (RMC ∼ 3) is compatible with clinically relevant NGAL elevations.^9,13,54^ Further improvements in CysC performance would require standard immunoassay optimization steps, including antibody pair screening, adjustment of bead functionalization, and flow conditions to match target-specific binding kinetics. The double-marker LMBA architecture is designed to support such independent optimization of assay parameters in each parallel channel without affecting other targets.

Using the double-marker LMBA, we analyzed plasma samples from mice during the second postnatal week. Sporadic CysC 10 μm beads were observed in NGAL channels (0.3% ± 0.8%), while only minimal contamination of NGAL 7 μm beads was detected in CysC channels (6% ± 8%). The low cross-contamination of reagents confirms the reliability of the double-marker LMBA spatial multiplexing approach, enabling the readout of both assays without the need for distinct fluorophores. This simplifies the optical setup and acquisition procedure, allowing the system to be optimized in terms of background suppression and excitation intensity for a single fluorophore. In contrast, the use of multiple fluorophores typically introduces additional sources of variability, as fluorophores differ in quantum yield, photostability, brightness, labeling efficiency, and environmental sensitivity, which can complicate quantitative comparisons between assays.^20^

Double-marker LMBA testing of plasma samples confirmed NGAL elevation in cKO-Ksp at P14 but not in *Bicc1* ^–/–^ at P10 (Figure 5e), as observed with single-marker tests (Figure 4c). Plasma CysC levels (Figure 5f) remained at low basal levels in WT controls, while spiking sporadically in mutant mice, with some PKD mice exhibiting a 10-fold increase compared to WT baselines. Elevated CysC, associated with impaired glomerular filtration rate, may identify PKD mice with a severe phenotype approaching kidney failure. Despite the limited cohort, LMBA multimarker quantification maintained an excellent correlation with ELISA, demonstrating the potential of this rapid testing approach to provide complementary information on renal state from only one microliter of sample.

## Conclusion

Near-patient quantification of multiple kidney biomarkers outside centralized laboratories could enable timely assessment of kidney function and improved patient stratification in both acute and chronic kidney disease. This study introduced the first rapid test for quantifying NGAL and CysC with minimal user intervention. The LMBA platform performs bead-based immunoassays within 20 min using an automated protocol that requires only postdilution sample dispensing. A portable cassette integrates a microfluidic unit with two independent paper components, establishing a lateral flow capillary circuit.

Across more than 100 independent assays, the LMBA system demonstrated robustness and consistency in generating quantitative data using a one-step workflow, benchmarked against ELISA. The analytical performance of the LMBA in PKD mouse matrices—including dynamic range, accuracy, precision, and analytical resolution—supports quantitative measurements across biomarker levels comparable to those observed in clinical AKI and CKD, demonstrating compatibility with clinically relevant concentration ranges and quantification requirements.

As a direct follow-up to the results obtained on PKD progression in mouse models, the LMBA’s low sample volume requirement will enable longitudinal monitoring via repeated sampling without the need for animal sacrifice at each time point. More broadly, the integration of LMBA with minimally invasive blood-collection techniques, such as fingerprick microsampling, suggests promising applications in emergency settings for AKI diagnosis and in decentralized facilities for longitudinal monitoring of PKD and other CKDs. In its current implementation, the LMBA is intended for operation by trained personnel in decentralized or near-patient settings, where limited sample preparation remains compatible with the workflow and quantitative biomarker outputs require clinical interpretation for patient stratification.

The LMBA platform offers a versatile and scalable workflow that readily translates to other biomarkers. The use of beads provides broad flexibility for surface chemical modification and biofunctionalization.^22^ Minimal reagent volumes enable rapid, cost-effective screening of various assay reagents and biochemical conditions. Following antibody pair selection, target-specific binding kinetics can be optimized by tuning flow rates via absorbent pad functionalization. Overall, the system’s modularity enables independent adjustment of parameters for reagent storage and resuspension, assay binding and signal readout, and flow rate and total sample volume. Looking ahead, the biomarker panel that can be simultaneously quantified by LMBA could be expanded to a few tens by combining spatial multiplexing with multiple bead sizes, positioning this solution as a flexible platform for rapid, multianalyte clinical diagnostics.

## Supplementary Material











## Data Availability

The data sets generated during and/or analyzed during the current study are deposited in Zenodo (doi: 10.5281/zenodo.21035299). The repository includes MATLAB codes for image processing and RMC calculation.

## Notes

Impact of running buffer composition on homogeneous bead-based assay (HBA) signal; image processing workflow for LMBA fluorescence quantification; wafer-level CAD layout and microfabrication process flow for hydrodynamic chip fabrication; endogenous NGAL and CysC levels in mouse urine and plasma samples; concentration ranges of NGAL and CysC in diluted samples for LMBA and ELISA quantification; evaluation of biological matrix effects and recovery rates in diluted urine and plasma; effect of endogenous interferents on NGAL homogeneous bead-based assay response; sample pad functionalization and reagent preloading strategy; stability of dry-stored assay reagents in the sample pad; optimization of dAb concentration and cAb-bead blocking conditions; temporal stability and batch-to-batch reproducibility of capillary flow in wax-functionalized absorbent pads; effect of absorbent pad wettability on LMBA end point signal; reference NGAL levels in different mouse strains; correlation between LMBA and ELISA quantification; precision analysis of LMBA and ELISA measurements; and LMBA dose–response curve for cystatin C quantification (PDF)
Time-lapse fluorescence binding kinetics (Video V1) (MP4)
LMBA operation (Video V2) (MP4)
Absorbent pad flow under different paper functionalizations (Video V3) (MP4)
Spatial multiplexing for double-marker detection (Video V4) (MP4)
