# Supplementary material for: Self-Contained Lateral-Flow Microfluidic Bead-Based Assay for Rapid Quantification of Early-Stage Kidney Biomarkers
Source: Anal Chem. 2026 Jul 1;98(27):20204–17. doi: 10.1021/acs.analchem.6c01144 (PMC13373927; doi:10.1021/acs.analchem.6c01144)
Supplement: Supplementary file 1 [file ac6c01144_si_001.pdf]

Supporting information for publication

Self-contained lateral-flow microfluidic bead-based assay for rapid quantification of early-stage kidney biomarkers

*Gloria Porro[1], Micaela Siria Cristofori[1], Céline Gagnieux[2], Elia Pennati[1], Pierre-Emmanuel Thiriet[1,3], Daniel Constam[2], and Carlotta Guiducci[1,**]*

[1] Laboratory of Life Science Electronics, École polytechnique fédérale de Lausanne, Lausanne, 1015, Switzerland

[2] Laboratory of Developmental and Cancer Cell Biology, École polytechnique fédérale de Lausanne, Lausanne, 1015, Switzerland

[3] Rea Diagnostics SA, Lausanne, 1015, Switzerland

*carlotta.guiducci@epfl.ch (corresponding author)

Table of contents

The following files are available free of charge.

Impact of running buffer composition on homogeneous bead-based assay (HBA) signal; image processing workflow for LMBA fluorescence quantification; wafer-level CAD layout and microfabrication process flow for hydrodynamic chip fabrication; endogenous NGAL and CysC levels in mouse urine and plasma samples; concentration ranges of NGAL and CysC in diluted samples for LMBA and ELISA quantification; evaluation of biological matrix effects and recovery rates in diluted urine and plasma; effect of endogenous interferents on NGAL homogeneous bead-based assay response; sample pad functionalization and reagent preloading strategy; stability of dry-stored assay reagents in the sample pad; optimization of dAb concentration and cAb-bead blocking conditions; temporal stability and batch-to-batch reproducibility of capillary flow in wax-functionalized absorbent pads; effect of absorbent pad wettability on LMBA endpoint signal; reference NGAL levels in different mouse strains; correlation between LMBA and ELISA quantification; precision analysis of LMBA and ELISA measurements; and LMBA dose-response curve for cystatin C quantification (PDF).

Video demonstrations of time-lapse fluorescence binding kinetics, LMBA device operation, absorbent pad flow under different paper functionalizations, and spatial multiplexing for double-marker detection (MP4).

Impact of running buffer on LMBA signal

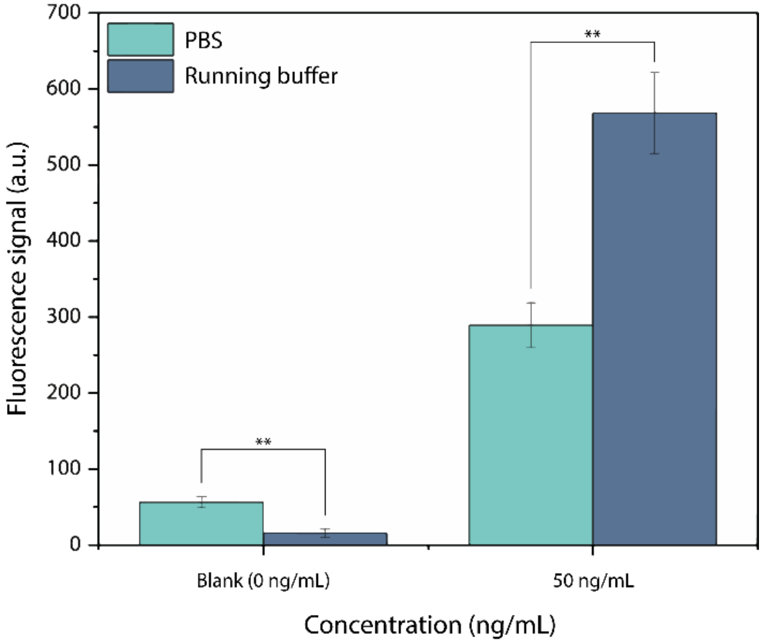

Figure S1. Homogeneous bead-based assay (HBA) buffer. Blank samples and solutions containing NGAL at 50 ng/mL were tested in PBS or the running buffer comprising 1% BSA and 0.05% Tween® 20 in PBS. Results represent the mean HBA fluorescence signal ± standard deviation from three independent assays per condition. Statistical significance was assessed using a two-tailed Student's t-test for independent samples, following verification of normality with the Shapiro–Wilk test (**$p \leq 0.01$).

Image processing for LMBA readout

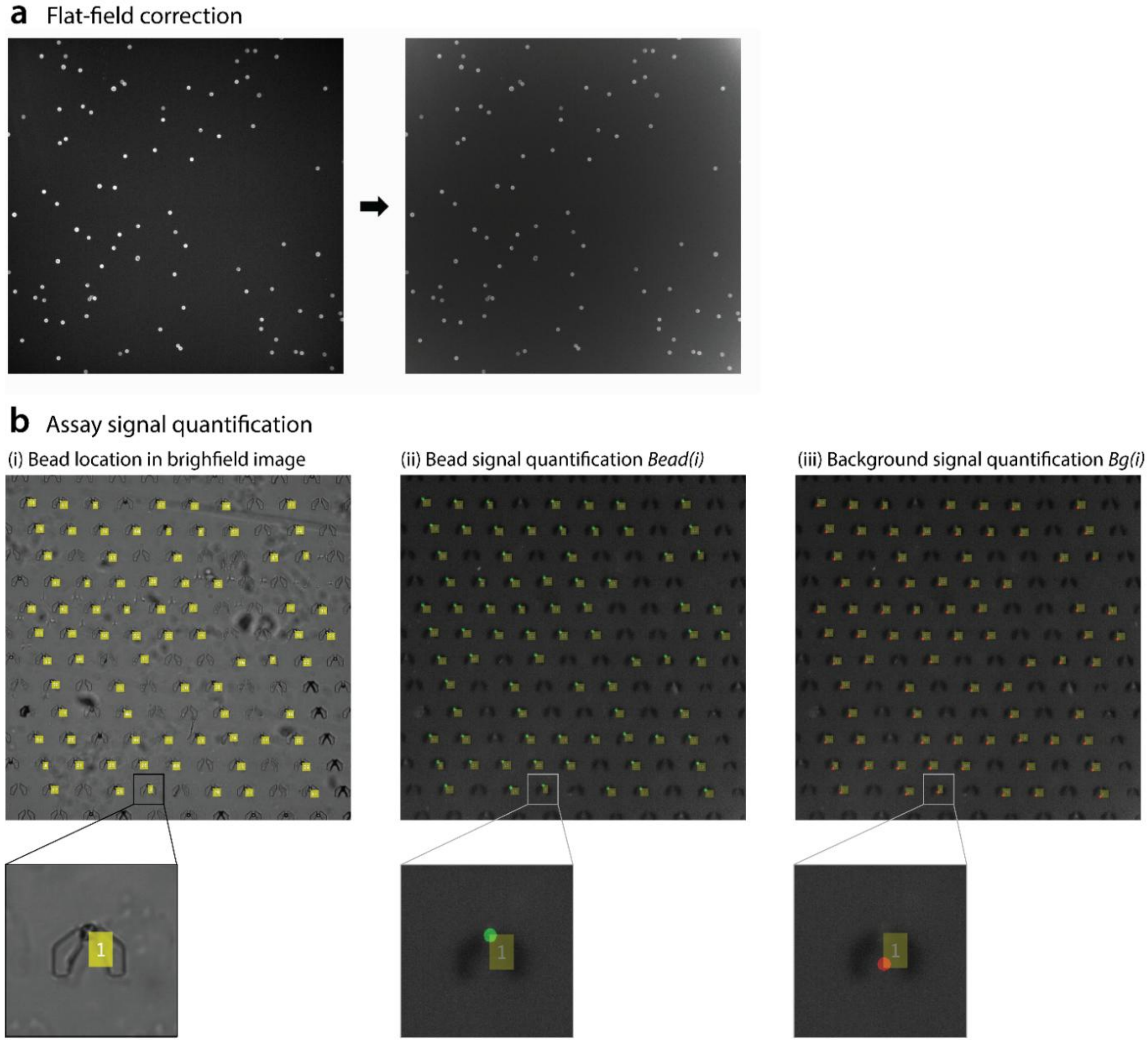

Figure S2. **Image processing workflow for fluorescence signal quantification in bead-based assays.** The image processing pipeline, implemented using a custom MATLAB script (refer to Zenodo, doi: 10.5281/zenodo.21035299, for details), is described as follows: **a.** Fluorescence images are first rescaled using flat-field correction to mitigate uneven illumination across the field of view. Representative fluorescence images from an NGAL HBA assay are shown. **b.** For the assay signal quantification (i) beads are identified as circular objects in the brightfield image; (ii)

the fluorescence intensity is quantified by measuring the bead signal, Bead(i), and the corresponding background signal, Bg(i), from the fluorescence image; (iii) the final assay signal is calculated as the average of the corrected intensity values: Average (Bead(i)-Bg(i)).

Wafer-level layout for microfabrication

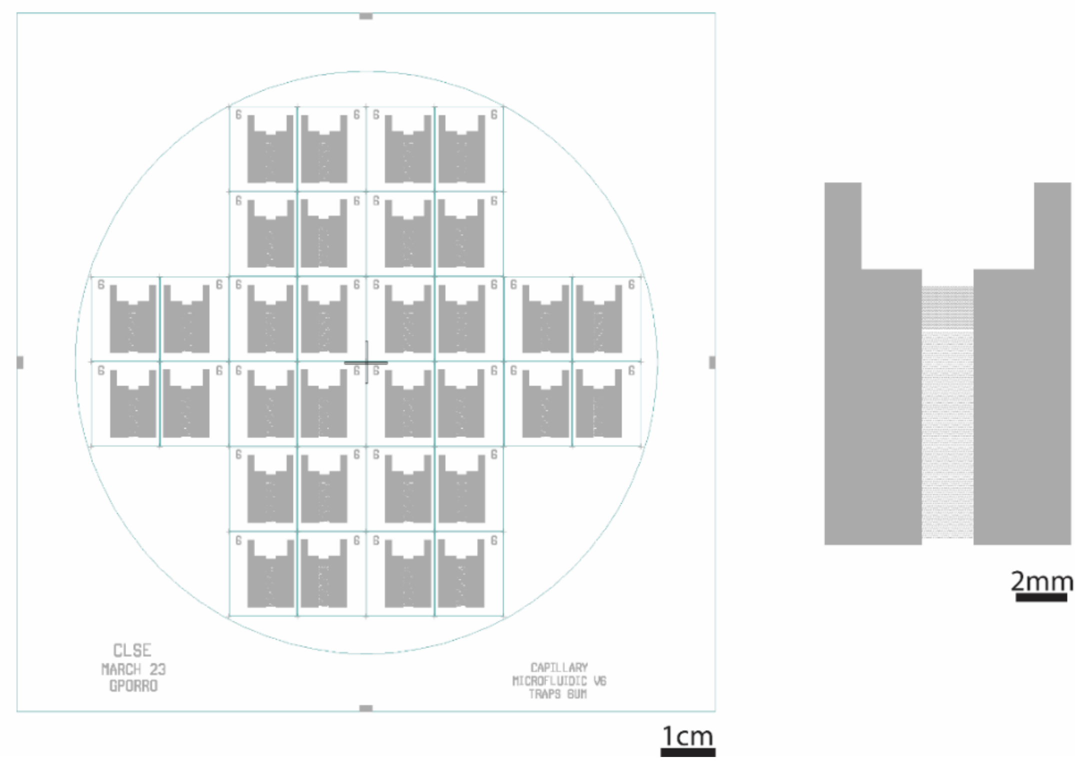

Figure S3. CAD layout for microfabrication. L-edit layout image for a 5-inch mask (left) and single hydrodynamic chip (right).

Microfabrication process flow

STEP	DESCRIPTION	EQUIPMENT	PARAMETERS
**1**	Surface cleaning and activation	Oxygen plasma (Gigabatch, PVA TePla America, LLC)	500 W, 4 min
**2**	Spin coating	Spin coater (LMS250, Sawatec, Switzerland)	SU8 Kayaku 3025 (Kayaku advanced materials)<br>Segment 1: time 5s, speed 500 rpm<br>Segment 2: time 5s, speed 500 rpm<br>Segment 3: time 21s, speed 2606 rpm<br>Segment 4: time 40s, speed 2606 rpm<br>Segment 5: time 1s, speed 3606 rpm<br>Segment 6: time 1s, speed 2606 rpm<br>Segment 7: time 5s, speed 2606 rpm<br>Segment 8: time 26s, speed 0 rpm
**3**	Soft bake	Hotplate	95°C for 12 min
**4**	Relaxation		Minimum 1 min
**5**	Exposure	UV exposure tool (MA6Gen3, Süss Microtech SE, Germany)	Hard contact, i-line filter, 170 mJ/cm^2
**6**	Post-exposure bake	Hotplate	Segment 1: 65°C for 1 min<br>Segment 2: 95°C for 3 min:20 s
**7**	Relaxation		Minimum 10 min
**8**	Development	Wet bench	Step 1: Place in PGMEA bath for 2 min<br>Step 2: Place in second PGMEA bath for 1min:20s<br>Step 3: Place in IPA bath for 1 min

Table S1. Microfabrication process. Detailed process flow for the microfabrication of the hydrodynamic chips.

Biomarker levels in the mouse cohort

	uNGAL	pNGAL	uCysC	pCysC
**Min (ng/mL)**	26	53	71	53
**Max (ng/mL)**	1539	1942	821	3820

Table S2. Endogenous levels of NGAL and CysC in mouse samples. This table summarizes the minimum and maximum endogenous levels of the biomarkers NGAL and CysC in urine (uNGAL, uCysC) and plasma (pNGAL, pCysC) samples from the mouse cohort under study. Levels were quantified at the time of collection using standard ELISA.

	uNGAL	pNGAL	uCysC	pCysC
**Min for LMBA quantification upon dilution (ng/mL)**	0.5	0.7	1.4	0.7
**Max for LMBA quantification upon dilution (ng/mL)**	30.8	24.3	16.4	47.8
**Min for ELISA quantification upon dilution (ng/mL)**	0.05	0.07	0.14	0.07
**Max for ELISA quantification upon dilution (ng/mL)**	3.08	2.43	1.64	4.78

Table S3. NGAL and CysC ranges in diluted mouse samples. Endogenous NGAL and CysC levels across the mouse cohort under study (Table S1) were scaled according to the respective dilution factors: 50-fold for LMBA and 500-fold for ELISA in urine (uNGAL, uCysC), 80-fold for LMBA and 800-fold for ELISA in plasma (pNGAL, pCysC). These scaled values define the actual concentration ranges that the two immunoassay methods must be able to quantify.

Impact of biological matrix effects on assay response

The homogeneous format (HBA) was employed to study the assay response in mouse samples. Matrix effects were first assessed through dose-response experiments using recombinant NGAL spiked in diluted biological matrices, as in **Figure S4a**. Urine and plasma samples with very low concentrations of endogenous NGAL, verified through ELISA, were used as blank matrices. For mouse urine, 60- and 50-times dilutions were adequate to generate dose-responses comparable to those observed in running buffer, with maintained sensitivity and dynamic range. Conversely, 60-fold diluted plasma samples resulted in a halving of sensitivity and a reduced dynamic range, with saturation occurring before 50 ng/mL. Matrix interference[1] primarily manifested as rotational effects varying with the analyte concentration, while translational effects were negligible, as indicated by comparable signals at low analyte concentrations[2,3]. Obtaining blood samples from mice before postnatal day 14 posed considerable challenges. Approximately 20% of plasma samples exhibited some degree of hemolysis, identified by red coloration.

To identify the dilution factors suppressing matrix effects while preserving the assay analytical performance, we quantified endogenous NGAL levels at increasing dilutions. We assessed recovery rates of HBA tests relative to reference values measured by ELISA (**Fig. S4b**). Confirming the milder interference posed by urine, dilutions ranging from 40 to 70 times all yielded acceptable recoveries, with variations below 10%. An 80-fold dilution was detrimental to the recovery rate, likely because the concentration approached the limit of quantification of the HBA. Ultimately, a 50-fold dilution was selected for urine samples to match the dynamic range of the LMBA. Plasma samples diluted up to 70-fold exhibited low recovery performance, while an 80-fold dilution enabled 100% recovery of the tested sample and was compatible with the LMBA dynamic range.

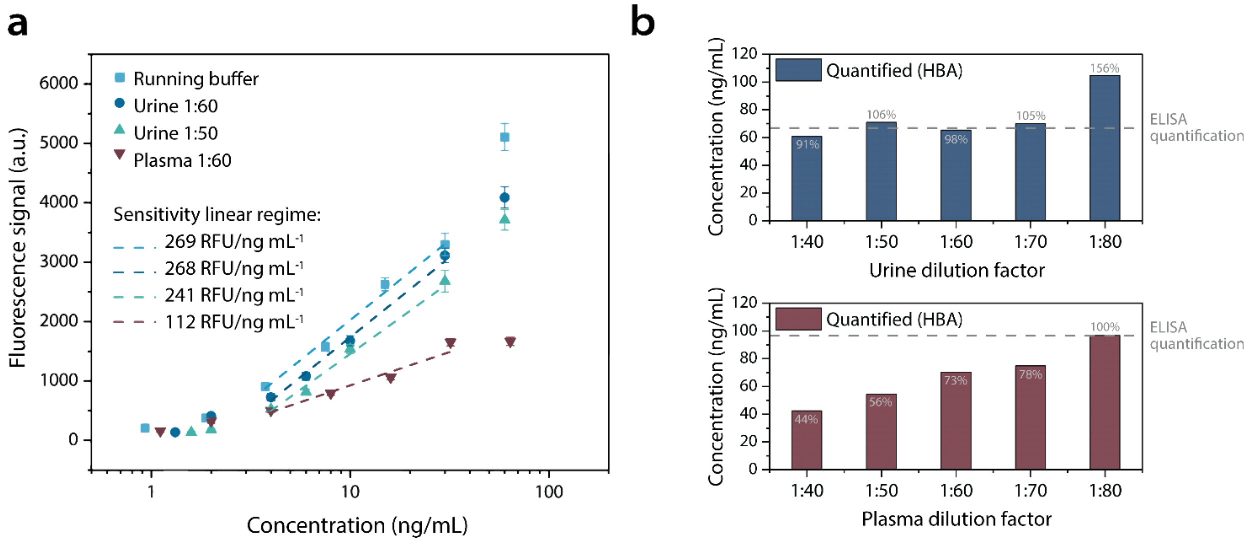

Figure S4. NGAL homogeneous bead-based assay (HBA) in mouse samples. a. HBA dose-response curves in different assay matrices with spiked recombinant NGAL. Data represent mean fluorescence signals (n=3 independent assays) with standard deviation as error bars. The assay sensitivities were calculated as the slopes of the fitted lines within the HBA linear regime (dashed lines). **b.** Endogenous NGAL levels quantified via HBA in urine (top, blue) and plasma (bottom, red) samples at increasing dilutions. The quantification was based on the HBA dose-response curve generated in running buffer (panel b). Recovery rates are expressed as percentages of HBA results relative to the reference levels measured by ELISA.

Interference studies

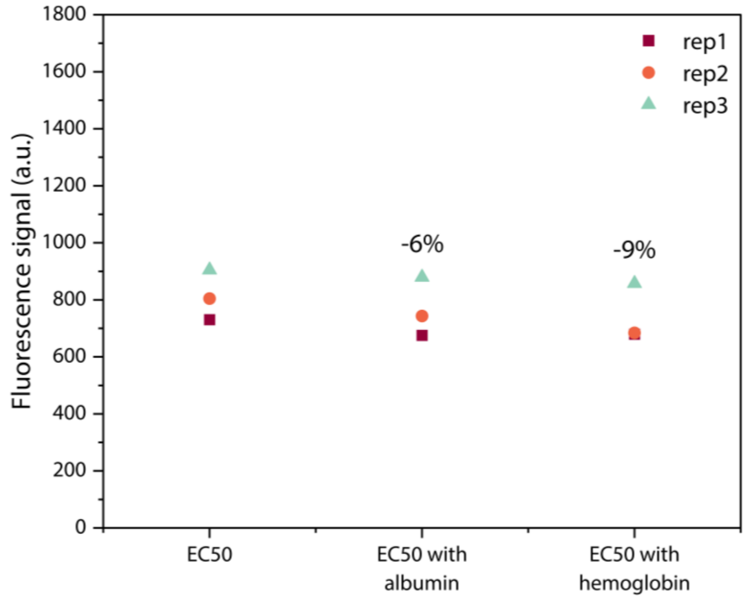

Figure S5. Effect of endogenous interferents on NGAL homogeneous bead-based assay (HBA) response. HBA fluorescence signals were measured at the NGAL EC50 concentration (17 ng/mL) in running buffer in the absence (control) or presence of representative endogenous interferents. Human serum albumin (4 mg/L) and hemoglobin (3.75 mg/dL), corresponding to clinically relevant pathological concentrations after application of the sample dilution factors used in this study, were used as interferents for urine and plasma matrices, respectively. Interference was quantified as the relative variation of the mean fluorescence signal compared with the interferent-free control.

Sample pad functionalization and storage

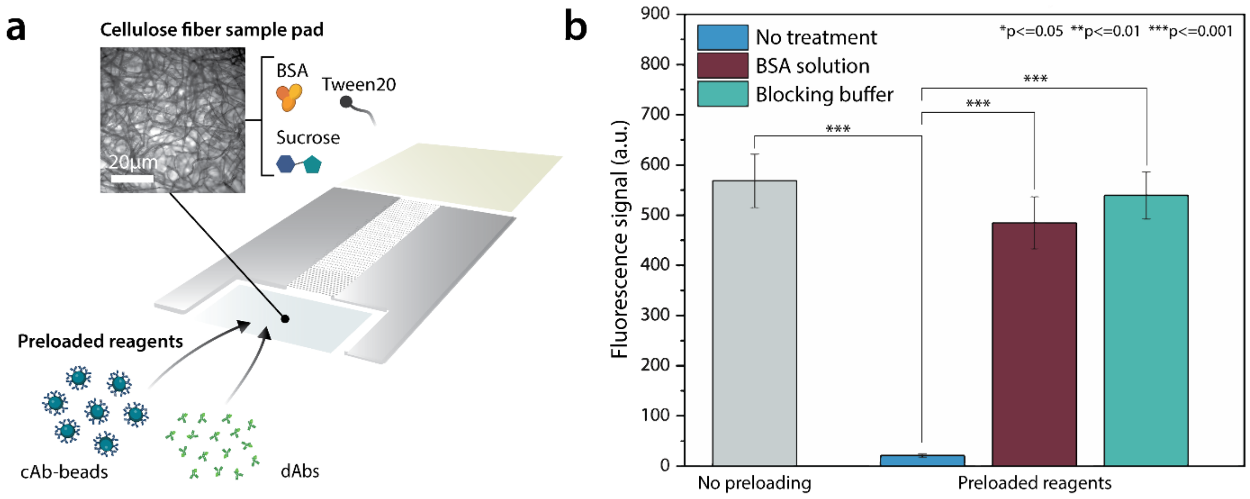

Figure S6. Blocking and preloading of reagents in the sample pad. **a.** Schematic representation of the LMBA system highlighting the cellulose fiber paper pad material. The cellulose fiber was treated with a blocking buffer (1% BSA, 5% sucrose, and 0.05% Tween® 20 in ultrapure water) to prevent unspecific binding of assay reagents. After blocking, assay reagents (cAb-beads and dAbs) were preloaded onto the sample pad. **b.** Fluorescence signals from homogeneous bead-based assays (bulk mixing in a tube) using a 50 ng/mL cystatin C solution in running buffer. The assays were performed with reagents in liquid form (no preloading) versus preloaded reagents (24 hours after preloading and drying) resuspended from sample pads treated as follows: untreated (blue), blocked with 1% BSA in water (red), or blocked with the complete blocking buffer (blue). Results are shown as the mean ± standard deviation (n=3 independent assays). Statistical significance was tested using one-way ANOVA followed by Tukey's test for multiple comparisons: nonsignificant comparisons are not displayed (*p≤0.05 **p≤0.01***p≤0.001).

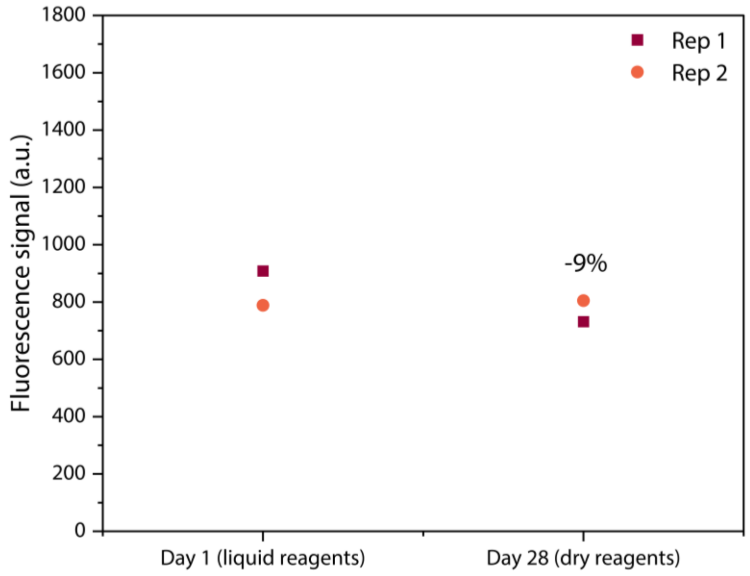

Figure S7. Stability of dry-stored assay reagents in the sample pad. LMBA fluorescence signals measured at the NGAL calibration point (16 ng/mL, near the assay EC50 of 17 ng/mL) in running buffer using freshly prepared reagents (cAb-beads and dAbs) in liquid form (day 1) or dry reagents preloaded in the sample pad and stored for 28 days at room temperature in a sealed pouch containing a silica gel desiccant. Signal decay was quantified as the relative variation of the mean fluorescence signal compared with the liquid-reagent control.

From homogeneous bead-based assay to LMBA: optimization of sensitivity and dynamic range

A key parameter influencing the assay signal is the concentration of detection antibodies (**Fig. S8a**). A 1X dAb solution, defined as the molar equivalent to NGAL at 50 ng/mL in a 50 µL sample volume, served as a baseline. 2X dAb concentration provided optimal performance, achieving a five-fold larger sensitivity than 1X in the linear regime, while reducing the LOD from 4 to 2 ng/mL. Although 3X extended the dynamic range to 100 ng/mL, it increased the LOD to 5 ng/mL and only marginally improved sensitivity (45%). In the selected 2X response, the 100 ng/mL signal was lower than 50 ng/mL, indicating a hook effect[2,4] for concentrations above the target NGAL range (**Table S3**).

Further decrease of the LOD was accomplished by minimizing the nonspecific binding on the surface of the beads. Increasing the BSA concentration in the blocking buffer from 1% to 10% and extending the blocking time from 2 hours to overnight resulted in a dose-response curve with a LOD of 100 pg/mL and unaffected the upper dynamic range (**Fig. S8b**). Optimized conditions were applied to the LMBA, which exhibited comparable fluorescence signals and dose-response sensitivities to the homogeneous assay condition. The LOD of the LMBA was determined to be 220 pg/mL, similar to the 100 pg/mL LOD of the HBA. LMBA devices provided a slightly higher background fluorescence, attributed to the adsorption of reagents on the PSA tape sealing the hydrodynamic chip.

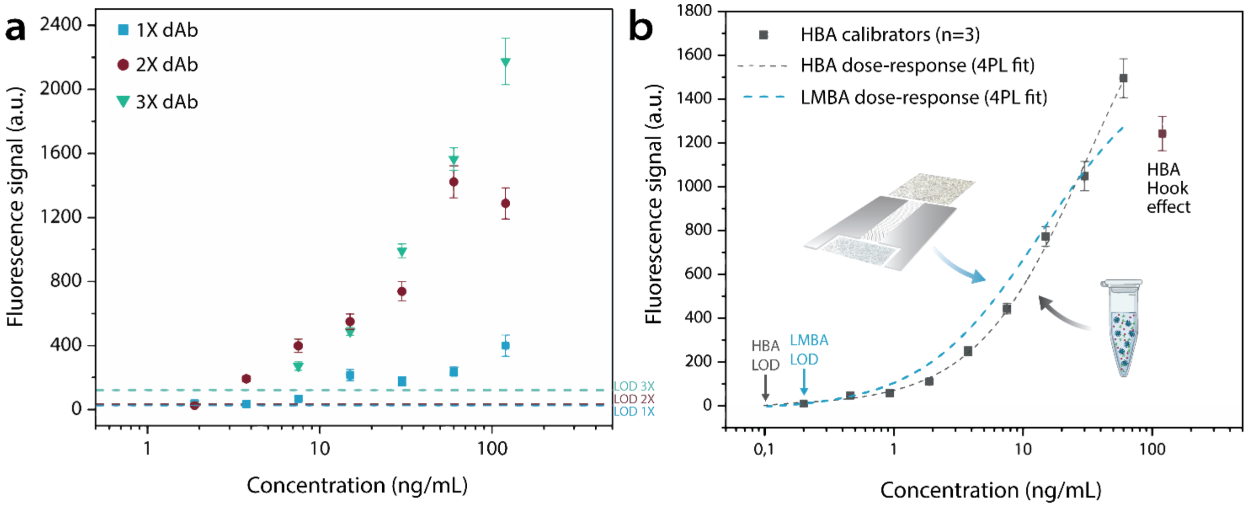

Figure S8. Dose-response performance optimization via homogeneous bead-based assays (HBAs). a. Dose-response curves for HBAs (bulk mixing of assay reagents in tubes) at different detection antibody (dAb) concentrations: 1X, 2X, and 3X, corresponding to dAb concentrations equal to, twice, or three times the NGAL molecules in a 50 µL sample at 50 ng/mL. Data represent mean fluorescence signals (n=3 independent assay replicates per condition) with standard deviation shown as error bars. Measured fluorescence signals were blank-subtracted. The limit of detection (LOD) was assessed as three times the standard deviation of signals from blank samples. **b.** Blank-corrected HBA dose-response curve in running buffer at the selected dAb concentration (2X) after improved blocking strategy. Data represent mean fluorescence signals (n=3 independent assay replicates per calibrator) with standard deviation as error bars, fitted using a four-parameter logistic (4-PL) regression model (gray dashed line). The calibrator point at 64 ng/mL was excluded from the regression range to exclude the hook effect artifact. The light blue dashed line represents the fitted dose-response curve for NGAL in running buffer quantified using the lateral-flow microfluidic bead-based assay (LMBA), data are shown in Figure 2a.

Flow rate stability and reproducibility

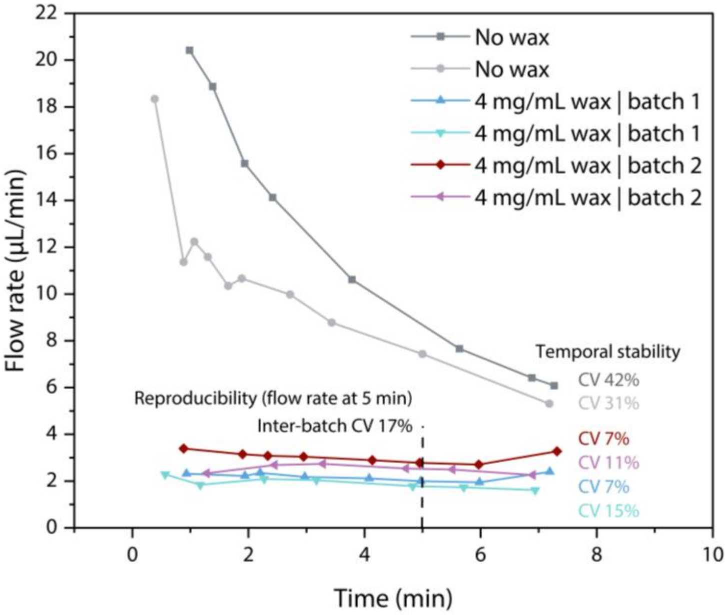

Figure S9. Temporal stability and batch-to-batch reproducibility of capillary flow in wax-functionalized absorbent pads. Time-dependent flow-rate measurements of untreated and wax-treated absorbent pads (4 mg/mL paraffin wax) during capillary-driven flow of 50 µL running buffer. Wax-treated pads were fabricated from two independent paraffin wax/toluene baths, with two pads tested per batch. Temporal flow stability was quantified by the coefficient of variation (CV) of the flow rate during the first 10 minutes of capillary flow for each pad. Fabrication reproducibility was assessed by comparing flow rates at 5 minutes across independently fabricated pads.

Effect of flow rate on LMBA endpoint signal

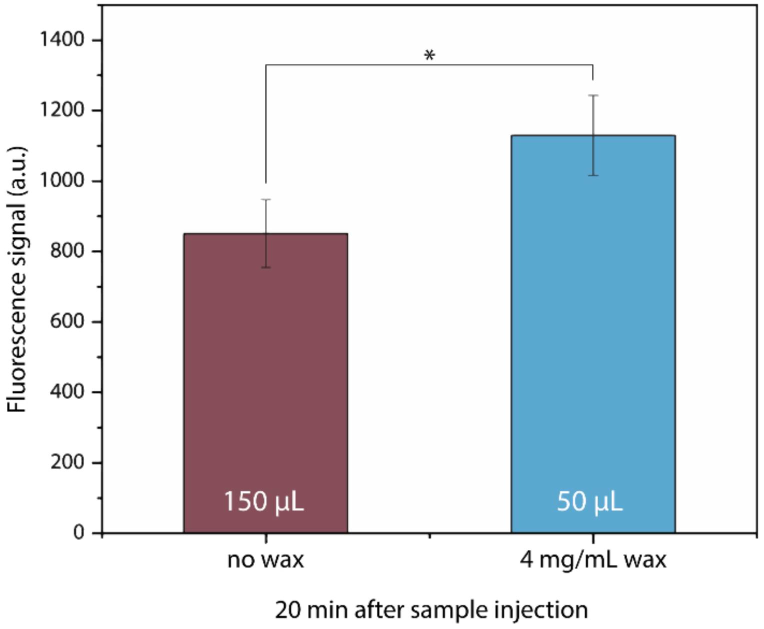

Figure S10. Impact of the absorbent pad wettability on the LMBA signal. LMBA fluorescence signal measurements of NGAL assays (16 ng/mL in running buffer) performed 20 minutes after sample dispensing (n=3 independent assays with over 50 beads conducted per condition; error bars represent standard deviation). Statistical significance was determined using a two-tailed Student's t-test for independent samples after the Shapiro–Wilk normality test (*$p \leq 0.05$).

NGAL levels in mice of different strains and genotypes

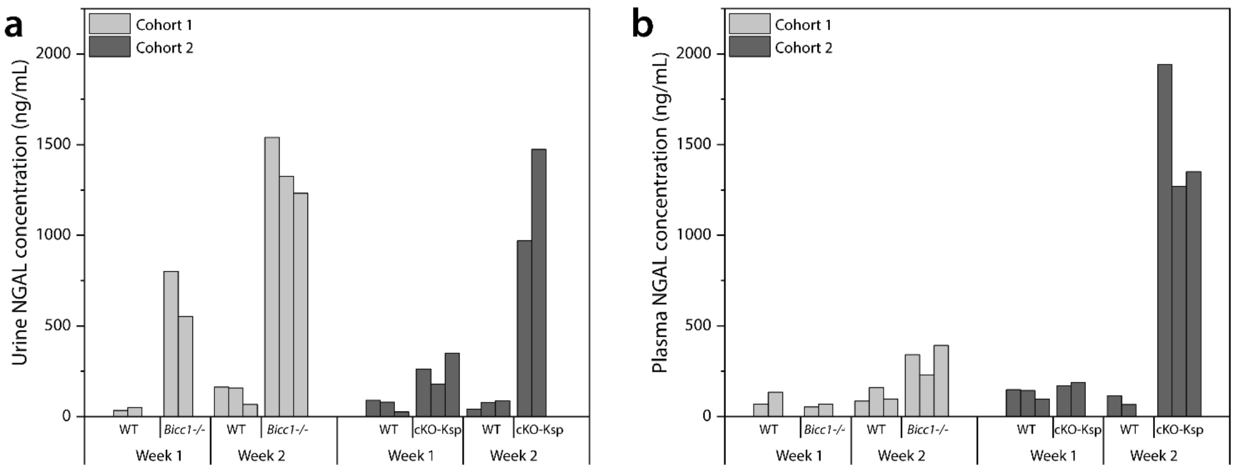

Figure S11. NGAL reference levels. ELISA measurements of NGAL levels at the time of collection of **a.** urine and **b.** plasma samples from the indicated *Bicc1* mutant mice and their WT control littermates.

Correlation between LMBA and ELISA measurements

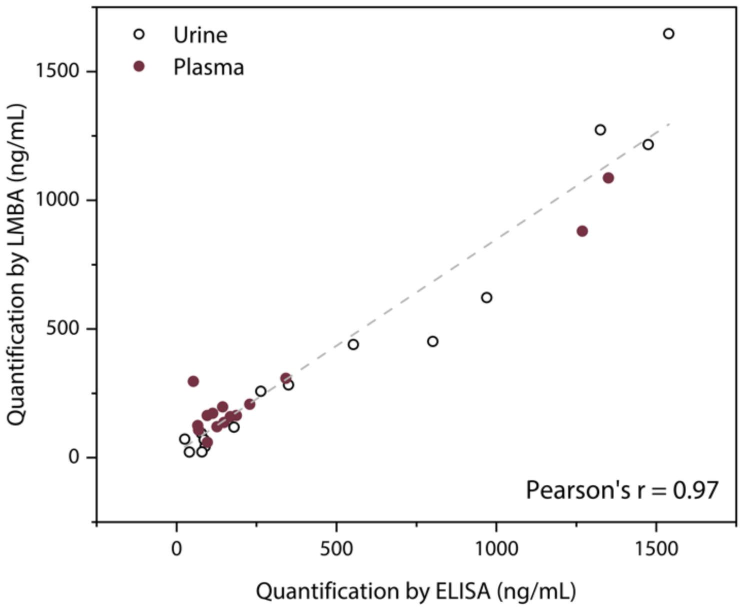

Figure S12. Correlation between LMBA and ELISA quantification of mouse samples. ELISA measurements (reference method) are plotted on the x-axis, while corresponding LMBA quantifications are displayed on the y-axis. The linear regression yielded an R-squared value of 0.93 and a Pearson's correlation coefficient (r) of 0.97.

Precision analysis of LMBA and ELISA measurements

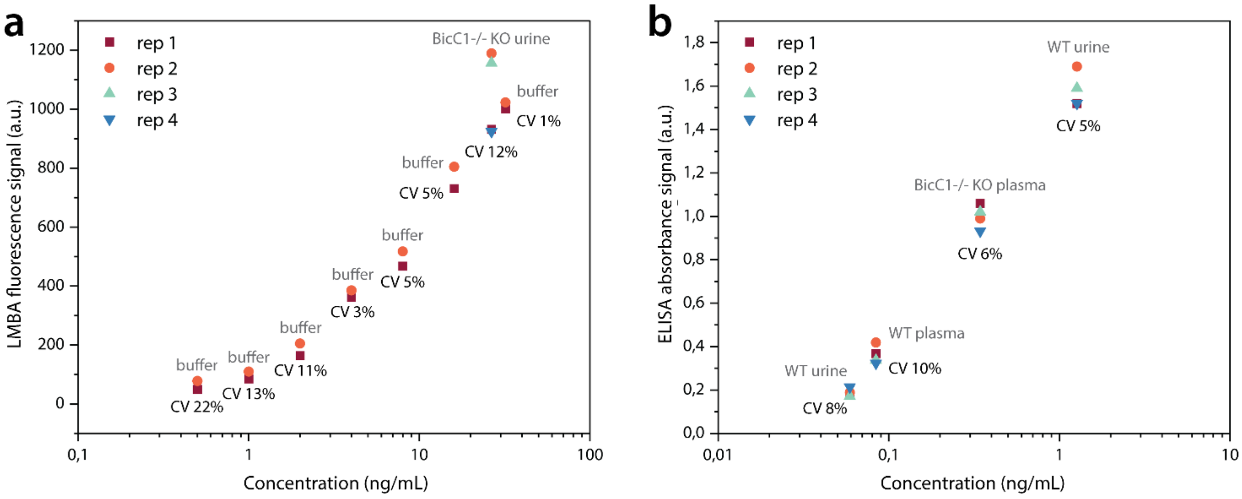

Figure S13. **Precision analysis of LMBA and ELISA**. The assay precision was evaluated using the coefficient of variation (CV), calculated as the ratio of the standard deviation to the mean of multiple signals from independent measurements of the same sample. **a.** The LMBA precision was evaluated by performing LMBA quantifications via the single-use devices; we considered replicates of calibrators in running buffer (n=2) and one urine sample from a *Bicc1*$^{-/-}$ mouse (n=4). **b.** Precision of ELISA was assessed by measuring replicates across separate microwells on the same plate (n=4) for four mouse samples: two distinct samples of wild-type (WT) mouse urine, one of WT plasma, and one *Bicc1*$^{-/-}$ plasma.

LMBA dose-response curve for CysC

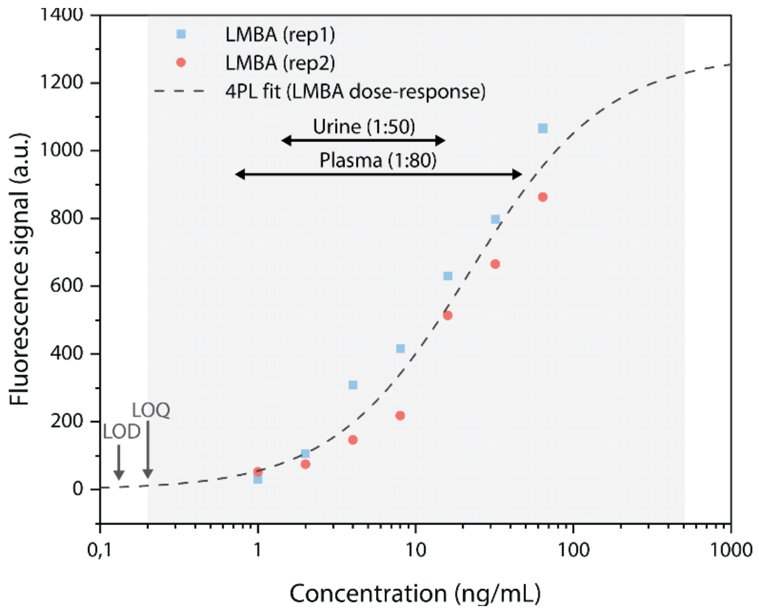

Figure S14. LMBA dose-response curve for cystatin C (CysC) quantification. Two independent replicates (rep 1,2) of standard dilution calibrators in running buffer were quantified using the LMBA platform. Fluorescence signals were blank-corrected based on the mean of five blank measurements (n=5) and fitted to a four-parameter logistic (4-PL) regression model, achieving an R-squared value of 0.95 (dashed line). The limit of detection (LOD) and limit of quantification (LOQ) were calculated as the concentrations corresponding to respectively three and five times the standard deviation of the blank (n=5). The dynamic range of the LMBA (gray-shaded area, ranging from the LOQ to the saturation point) is compared with the endogenous levels of CysC observed in urine and plasma samples from the analyzed mouse cohort, which were diluted 50-fold and 80-fold in running buffer, respectively.

REFERENCES

(1) Wild, D. The Immunoassay Handbook; Elsevier: Oxford, 2013. https://doi.org/10.1016/B978-0-08-097037-0.01001-0.

(2) Sturgeon, C. M.; Viljoen, A. Analytical Error and Interference in Immunoassay: Minimizing Risk. Ann. Clin. Biochem. 2011, 48 (Pt 5), 418–432. https://doi.org/10.1258/acb.2011.011073.

(3) Ellison, S. L. R.; Thompson, M. Standard Additions: Myth and Reality. Analyst 2008, 133 (8), 992–997. https://doi.org/10.1039/b717660k.

(4) Rey, E. G.; O'Dell, D.; Mehta, S.; Erickson, D. Mitigating the Hook Effect in Lateral Flow Sandwich Immunoassays Using Real-Time Reaction Kinetics. Anal. Chem. 2017, 89 (9), 5095–5100. https://doi.org/10.1021/acs.analchem.7b00638.
